# Supplementary material for: KIN: a method to infer relatedness from low-coverage ancient DNA
Source: Genome Biol. 2023 Jan 17;24:10. doi: 10.1186/s13059-023-02847-7 (PMC9843908; doi:10.1186/s13059-023-02847-7)
Supplement: Supplementary file 1 — Additional file 1: Supplementary figures. Figure S1. Overall schematic of the method showing the entire pipeline from processed bam files to final relatedness and IBD estimates. Figure S2. Comparison of fit with Beta-binomial and Binomial distributions. Figure S3. Overview of simulated dataset. Figure S4. Application of READ on Neandertal specimens from Chagyrskaya and Okladnikov Caves. Figure S5. Application of Identical KIN-HMM on a pair of identical individuals with low coverage (0.2x), and ROH tracts. Figure S6. Comparison of beta distributions estimated with the Identical KIN-HMM with and without variance constrained optimization of $\delta$δ parameters. Figure S7. Comparison of Beta distributions estimated with ROH-HMM with and without constrained emissions. Figure S8. False positive and true positive rates as a function of cutoff on log-likelihood ratio. Figure S9. Comparison of IBD estimation in control simulations and in presence of contamination, ascertainment or ROH at different coverages. Figure S10. Comparison of IBD states estimated for Chagyrskaya specimens using lcMLkin and KIN. Figure S11. Plots showing proportion of differences in windows along the genome for some pairs of relatives for which there is contradiction among KIN, READ and lcMLkin. Figure S12. Plots showing proportion of differences in windows along the genome for the first-degree relatives for which KIN differs from lcMLkin. Figure S13. False positive and true positive rates as a function of incorrect contamination estimates provided to KIN. [file 13059_2023_2847_MOESM1_ESM.pdf]

KIN: A method to infer relatedness from low-coverage ancient DNA

Divyaratan Popli, Stéphane Peyrégne, Benjamin M. Peter

November 19, 2022

Supplementary Figures

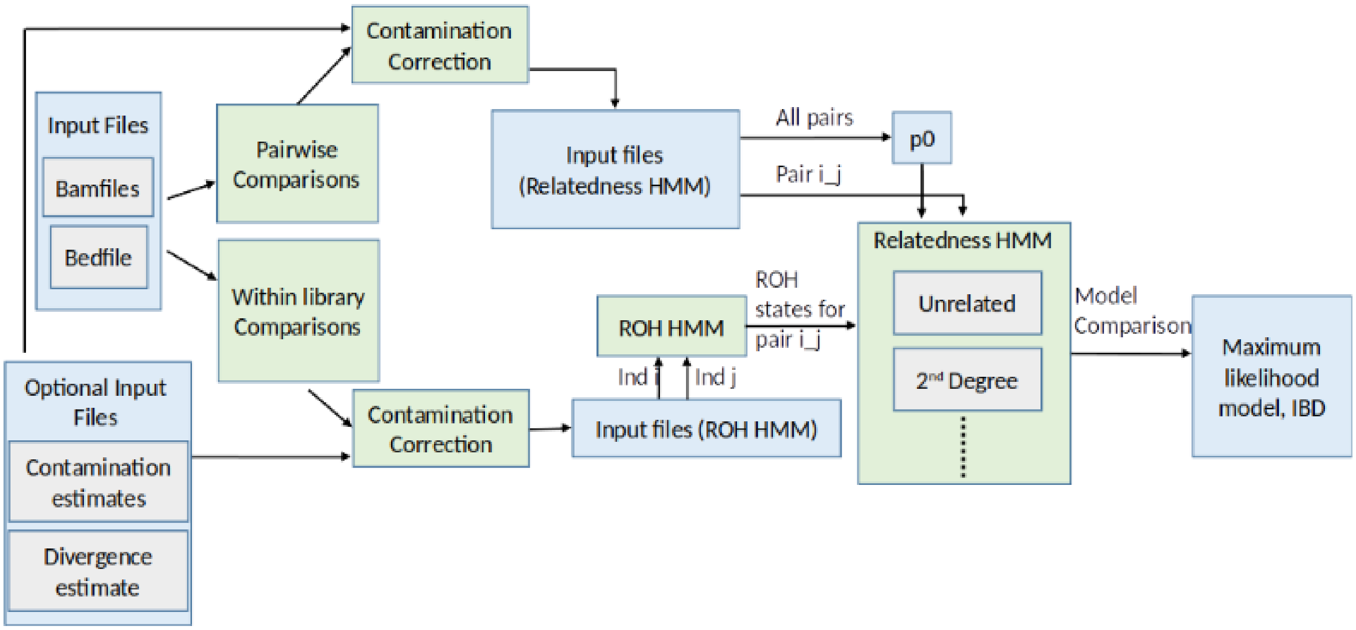

Fig S 1: Overall schematic of the method showing the entire pipeline from processed bam files to final relatedness and IBD estimates. Blue boxes show the data files, while green boxes represent scripts.

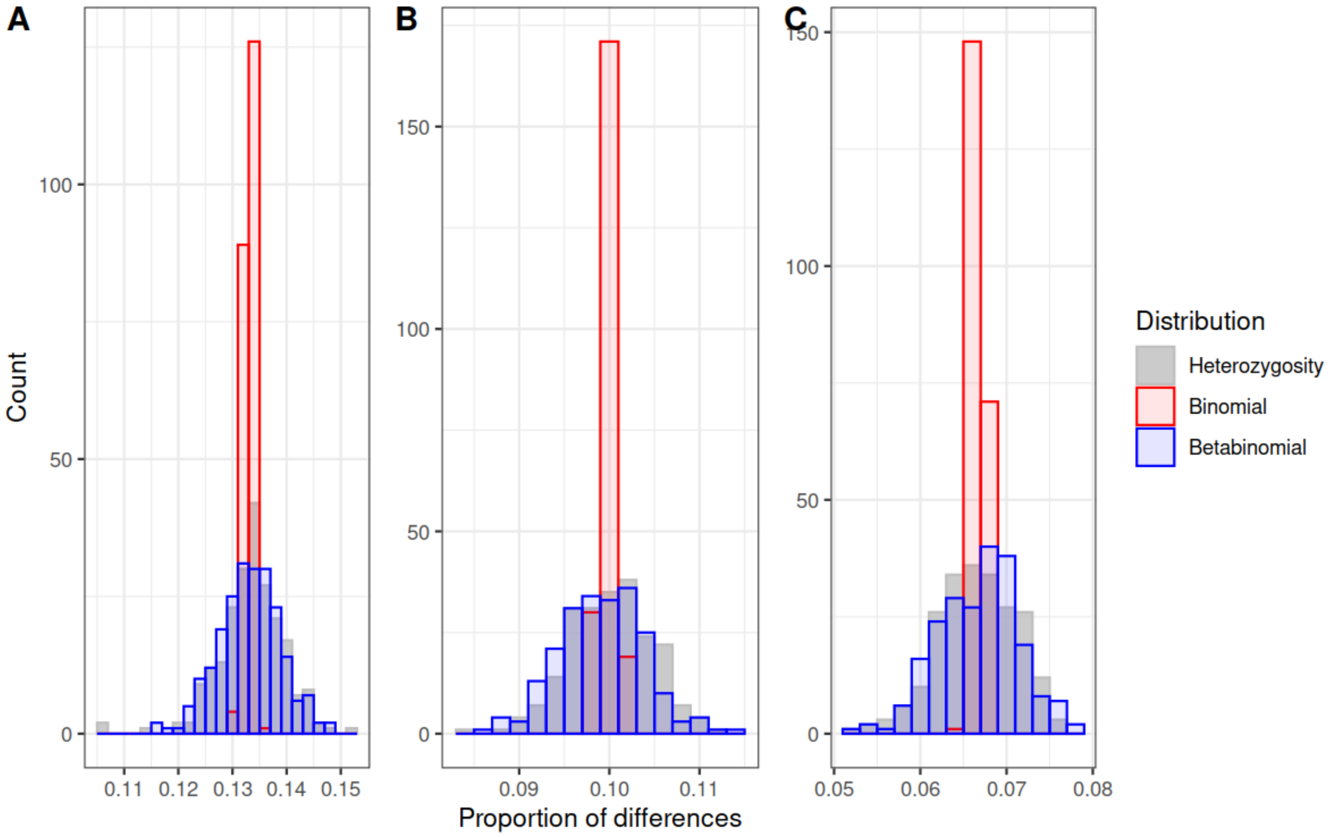

Fig S 2: Comparison of fit with Beta-binomial and Binomial distributions. Data is represented by the histogram of proportion of differences from all windows of a (A) unrelated, (B) Parent-Child, (C) Identical pair of individuals. Binomial parameter p is calculated from the data, while Beta-binomial parameters are estimated with corresponding KIN-HMM.

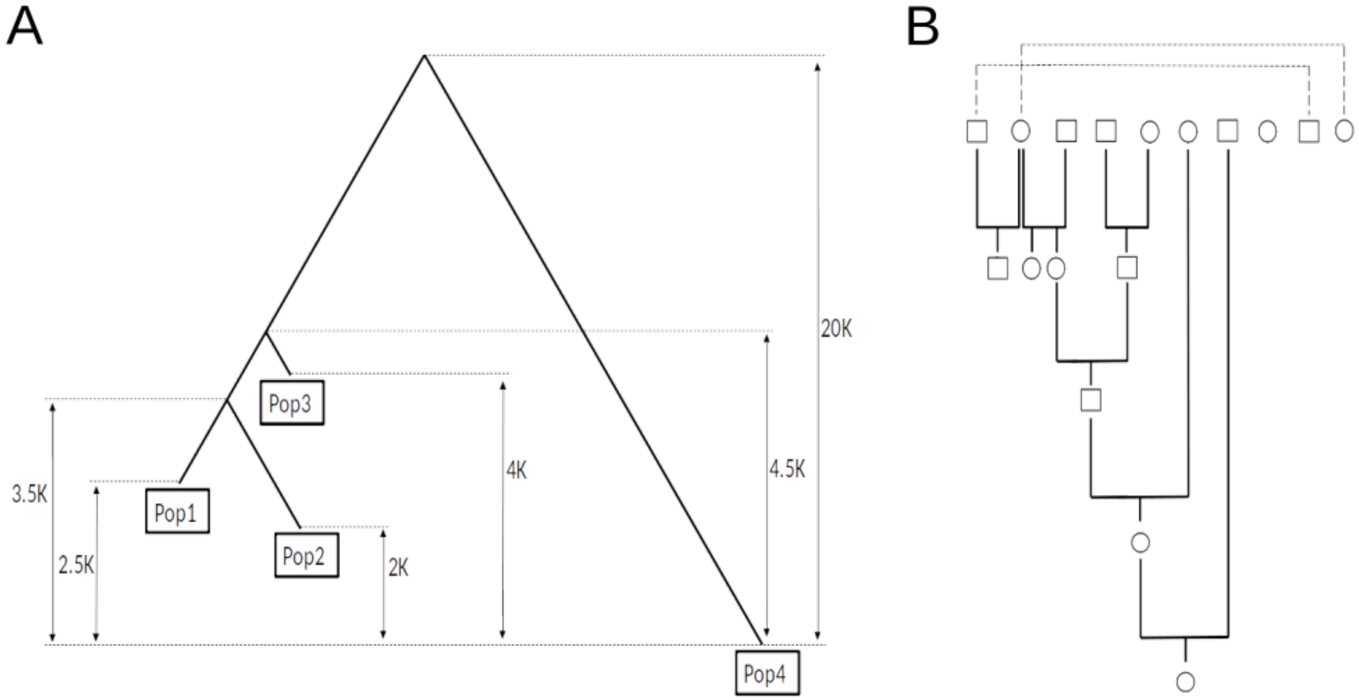

Fig S 3: Overview of simulated dataset. (A) We simulated four different populations with split times and sampling times (in generations) mimicking the populations of Chagyrskaya Neandertals, Vindija Neandertals, Altai Neandertals and present day Africans. We artificially mated individuals in Pop1 to create related individuals. We used Pop2 and Pop3 to introduce ascertainment bias in the data, while we simulated contamination from modern humans using Pop4. (B) In Pop1, we sampled 8 diploid unrelated individuals, and artificially mated some of them to make a pedigree with upto 5th Degree relatives. Circles represent females, and squares represent males. Dotted lines connect identical pair of individuals.

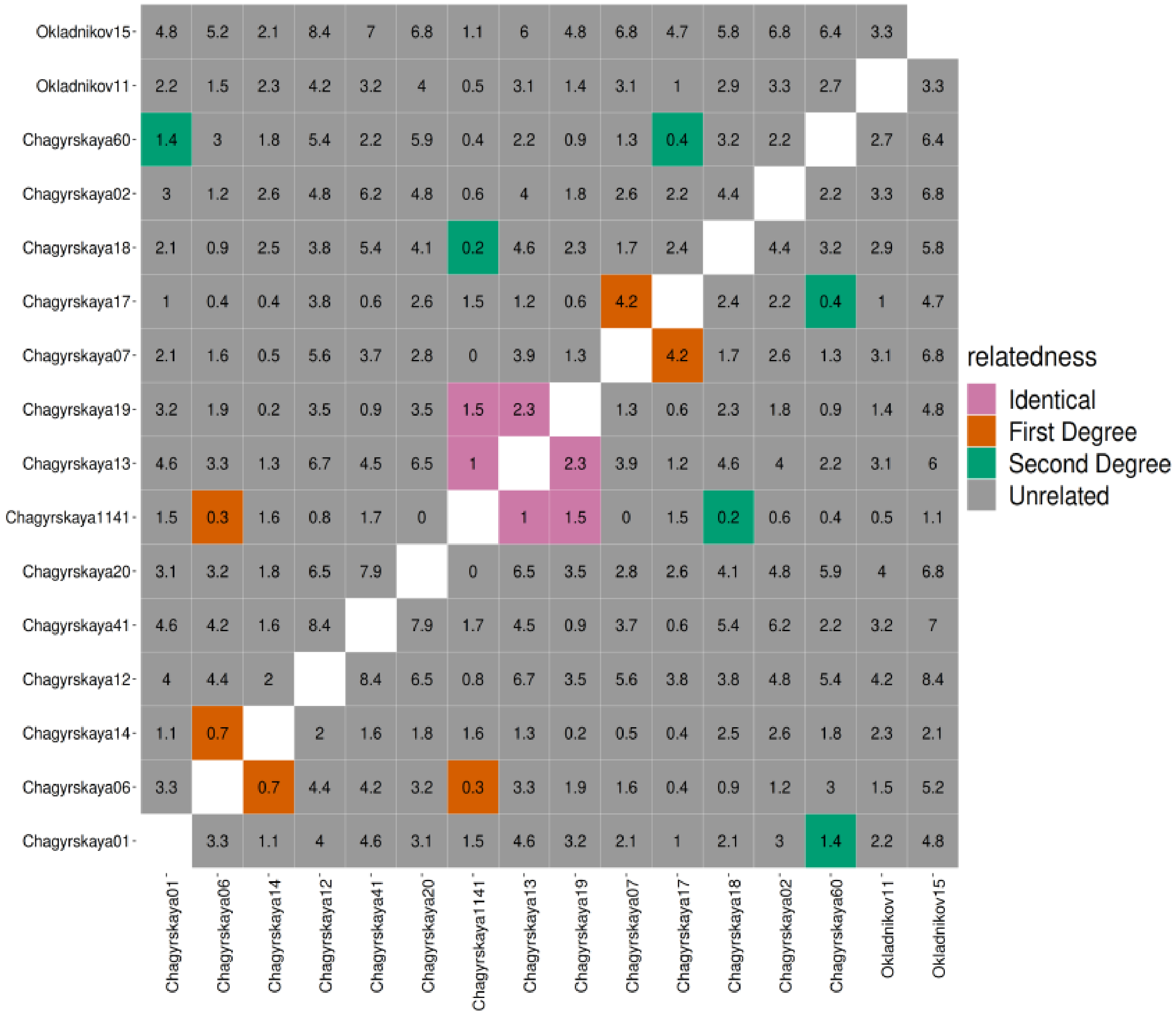

Fig S 4: Application of READ on Neandertal specimens from Chagyrskaya and Okladnikov Caves. Color of a square represents the relatedness, while the number denotes standard deviations away from the upper threshold (we show lower threshold for unrelated pairs since upper threshold is not available).

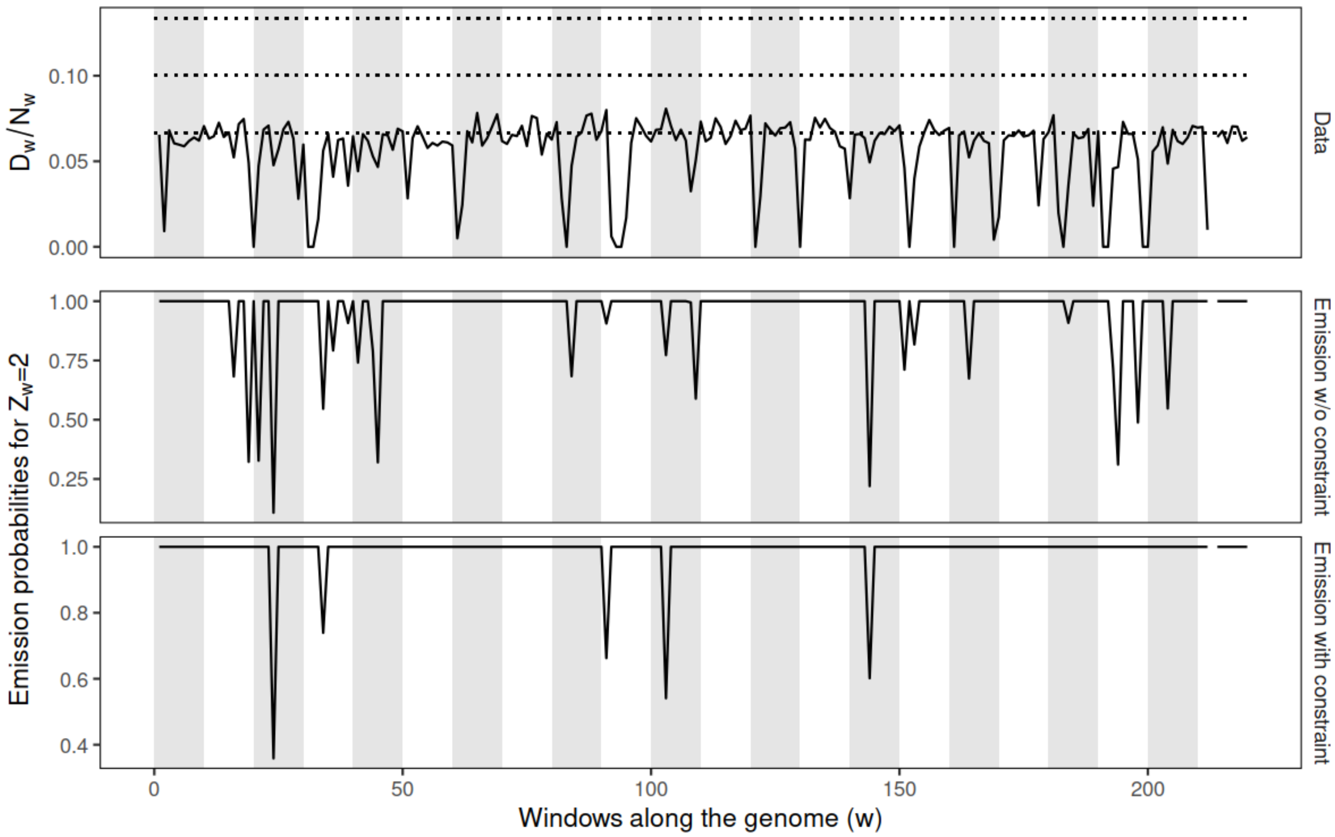

Fig S 5: Application of Identical KIN-HMM on a pair of identical individuals with low coverage (0.2x), and ROH tracts. Top panel shows pairwise proportion of differences is close to expectation, but dips in some windows due to presence of ROH tracts. Bottom two panels show $P(Data|Z_w = 2)$ without constraints and with constraints respectively.

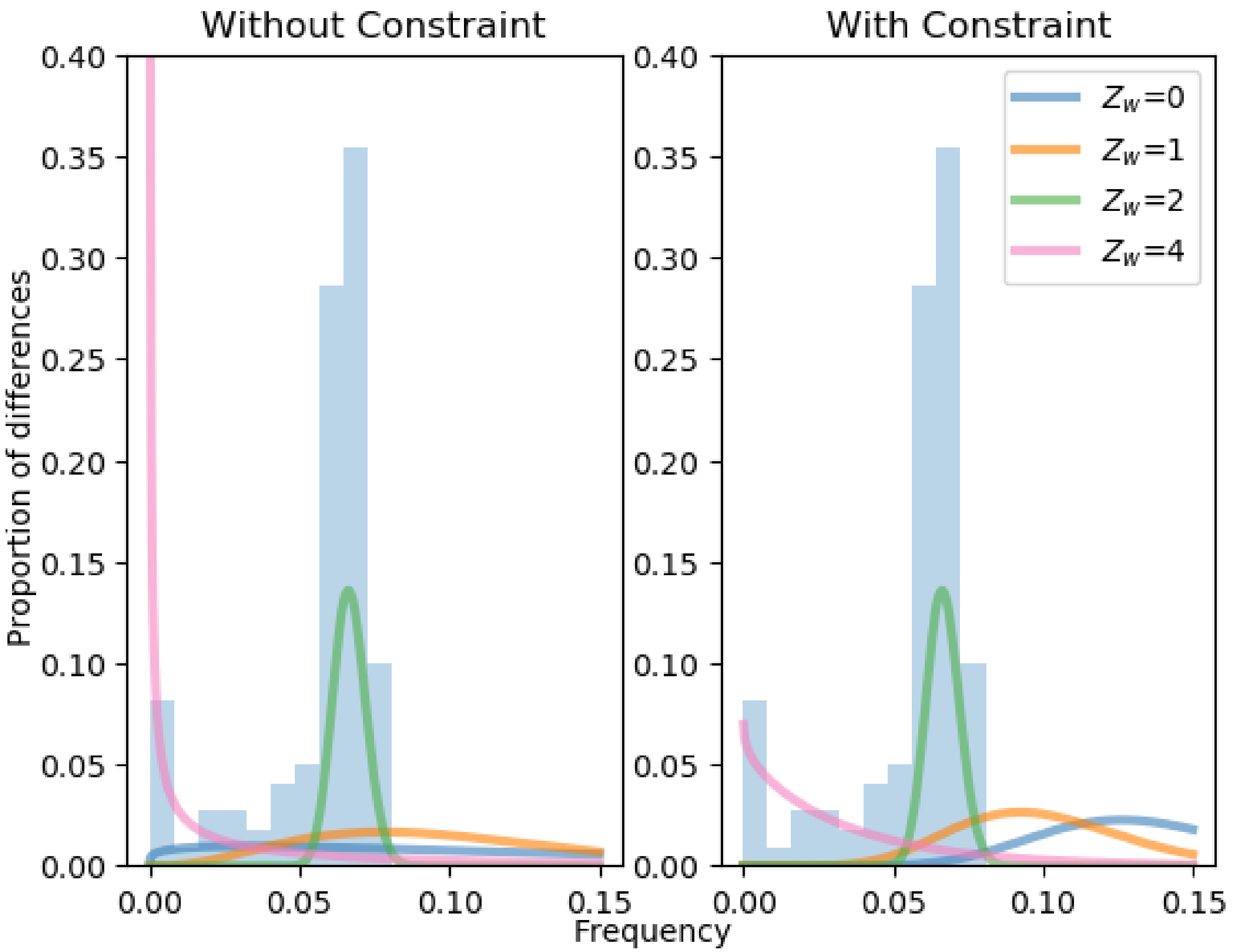

Fig S 6: Comparison of beta distributions estimated with the Identical KIN-HMM (left) without and (right) with variance constrained optimization of δ parameters.The histogram in each plot shows pairwise differences in genomic windows for identical individuals. Colored lines shows the Beta probability distribution for each i, where i is the index used for combinations of Z_w and H_w.

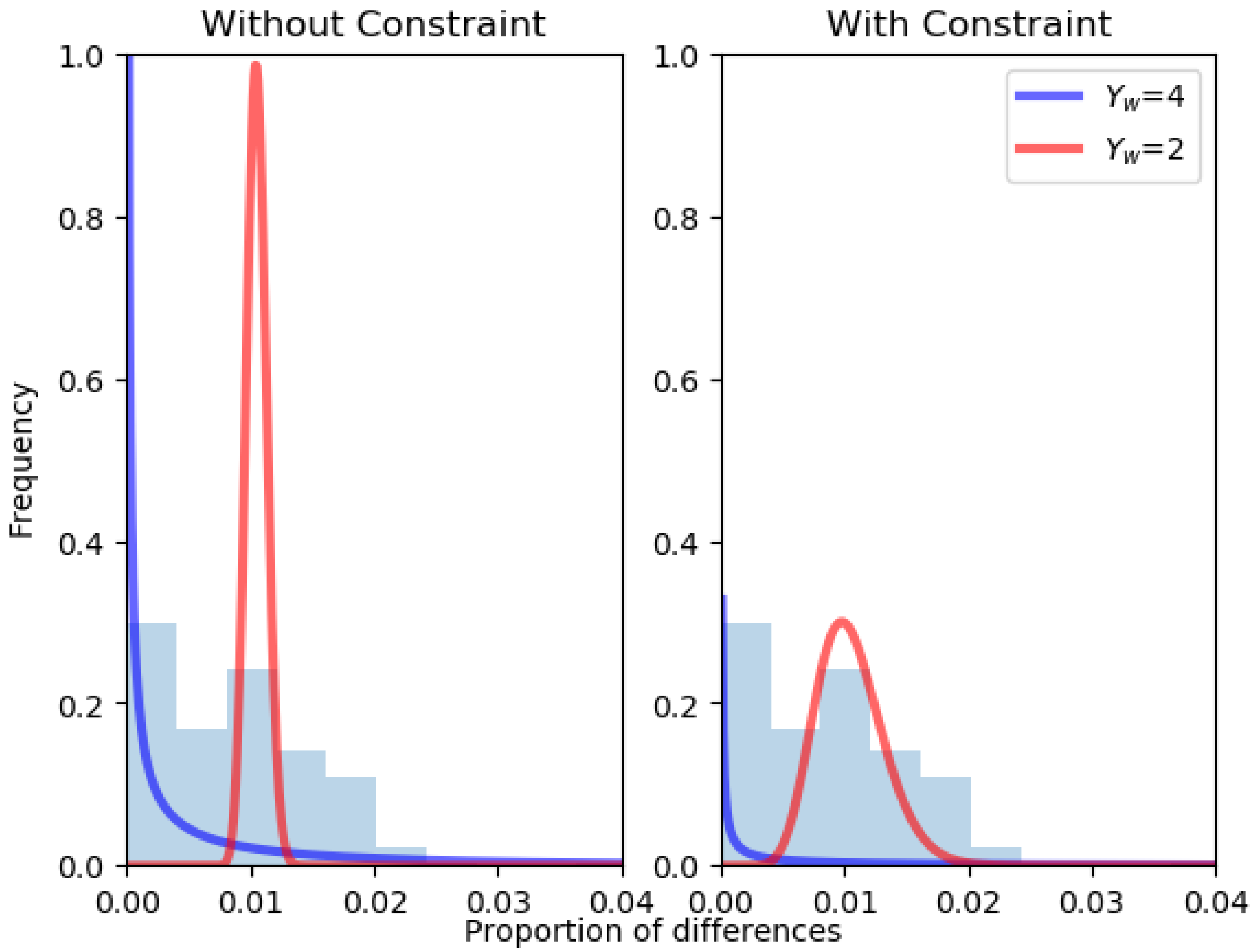

Fig S 7: Comparison of Beta distributions estimated with ROH-HMM (left) without and (right) with constrained emissions. The histogram in each plot shows pairwise differences in genomic windows for identical individuals. Colored lines shows the Beta probability distribution for each homozygosity state Y_w.

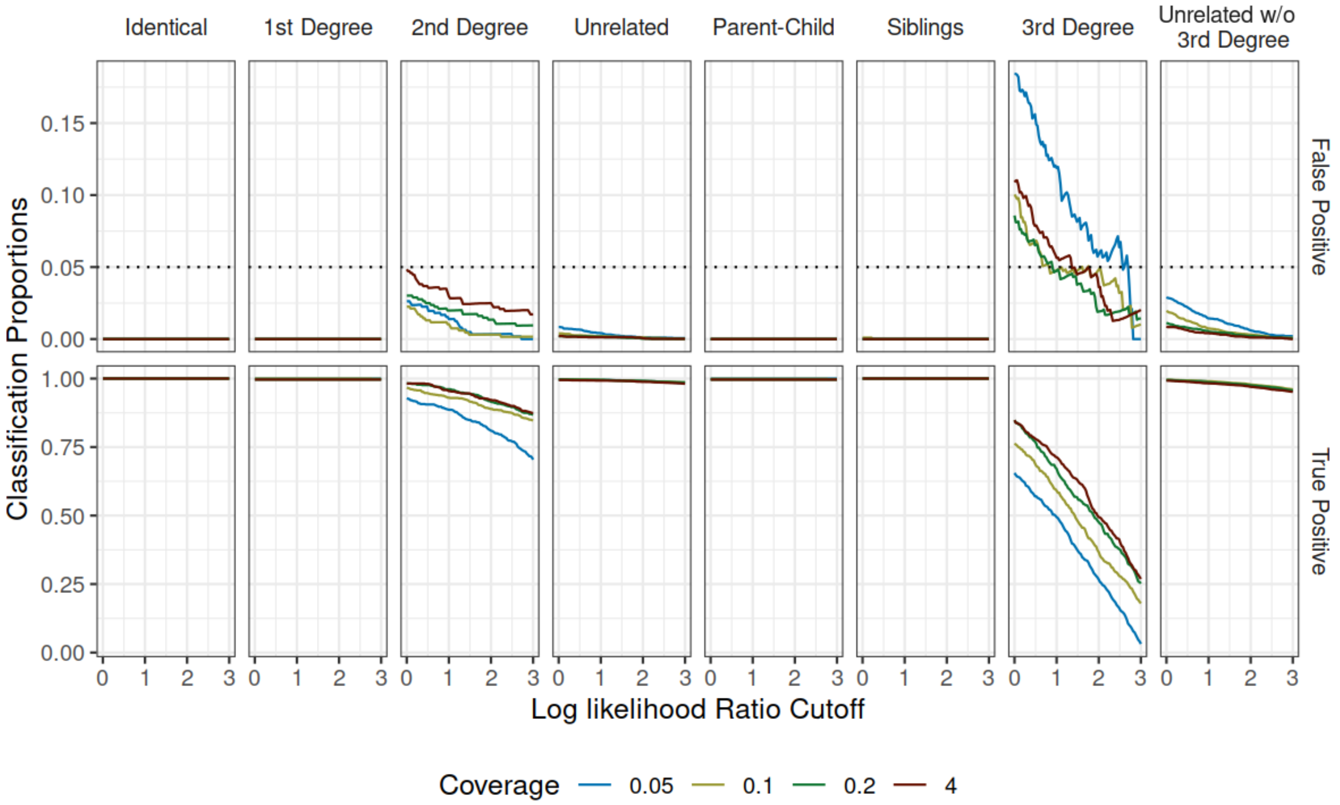

Fig S 8: False positive and true positive rates as a function of cutoff on log likelihood ratio. In top panel, dotted line represents 5%. 'Unrelated' label here refers to KIN performance results when all Unrelated, Fifth Degree, Fourth Degree, Third Degree pairs are labelled as Unrelated. 'Unrelated w/o 3rd Degree' refers to the performance results when 3rd Degree is classified separately from the unrelated individuals.

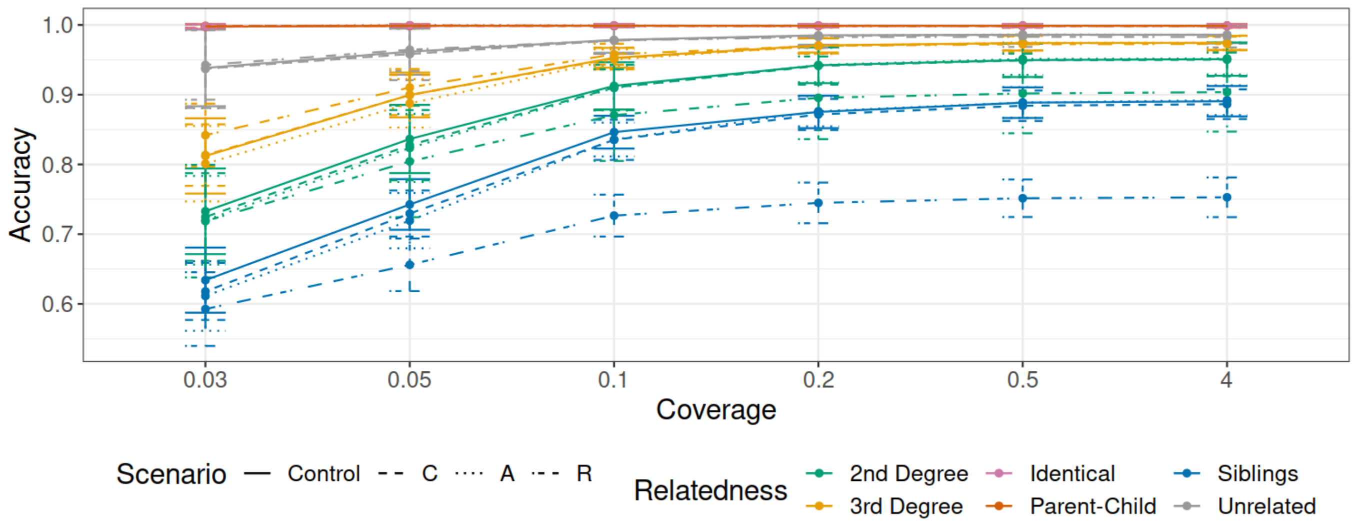

Fig S 9: Comparison of IBD estimation in control simulations and in presence of contamination, ascertainment or ROH (denoted in figure legend by Control, C, A and R, respectively) at different coverages. The y-axis shows accuracy calculated over 60 simulations for each relatedness case, for each scenario (control, C, A or R), and six different coverages shown on the x-axis. The error bars display one standard deviation from the mean. Here, accuracy corresponding to relatedness cases for Parent-Child and Identical individuals is always 1, and overlaps with each other.

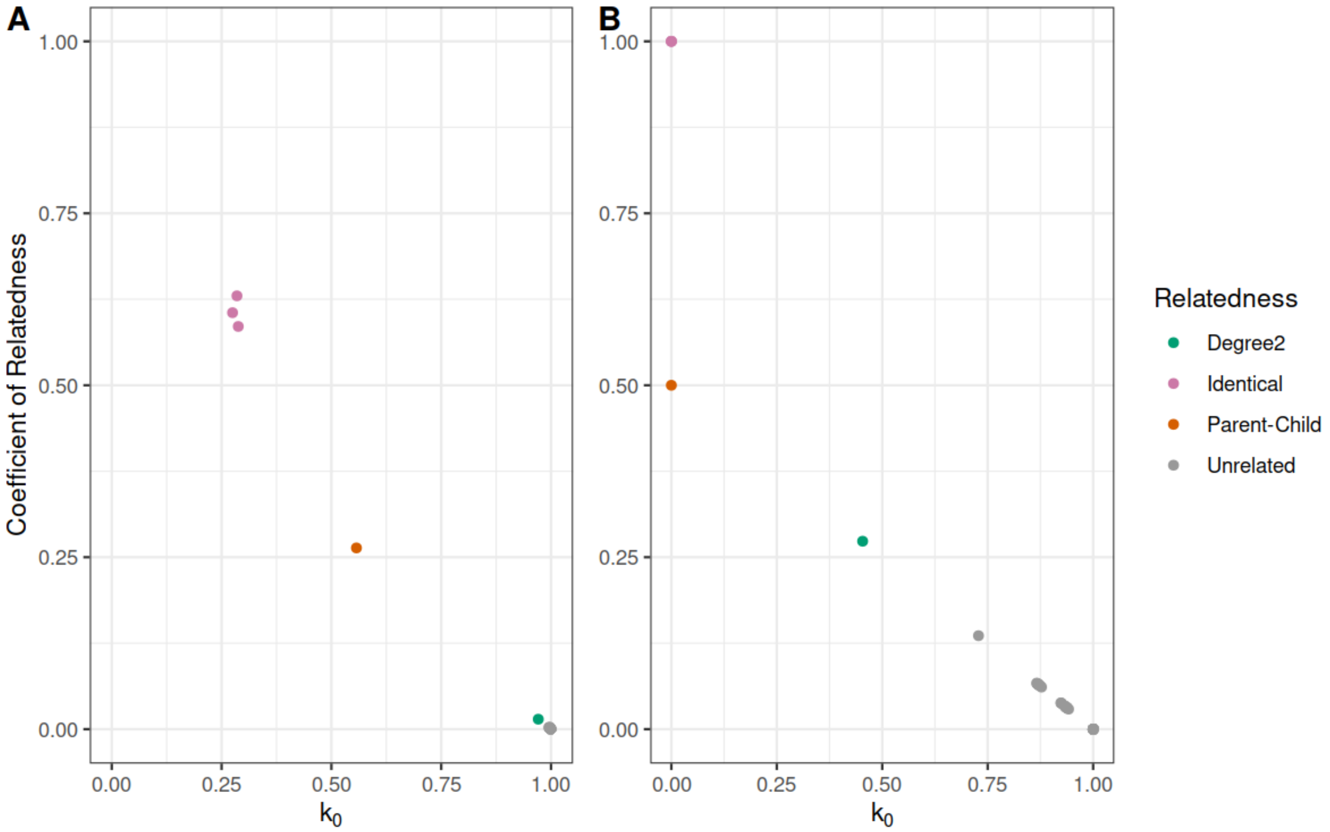

Fig S 10: Comparison of IBD states estimated for Chagyrskaya specimens using (A) lcMLkin and (B) KIN. Each plot shows relatedness coefficient (calculated as $k_2 + k_1/2$) on y-axis plotted against k_0 (proportion of genome with no chromosomes shared). The relatedness shown with different colors is estimated with both READ and KIN.

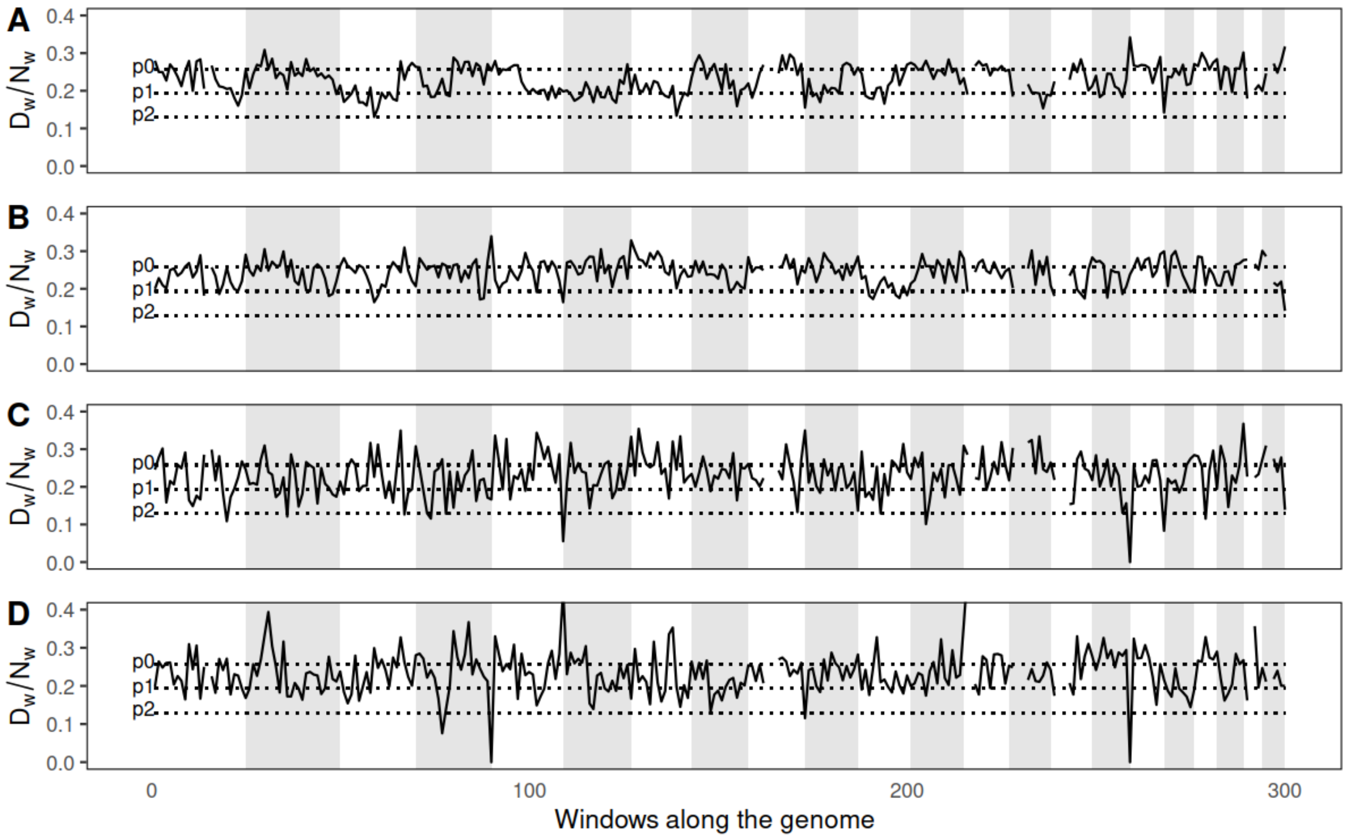

Fig S 11: Plots showing proportion of differences in windows along the genome for some pairs of relatives for which there is contradiction among KIN, READ and lcMLkin. (A) shows a case for which KIN and lcMLkin together disagree with READ, and (B) shows a case where KIN and lcMLkin differ, while READ does not classify between contradicting relatedness categories. (C) and (D) show the two unresolved pairs for which all three methods disagree. Note that we expect to see proportion of differences for second degree to be close to p_0 in 50% windows, and p_1 in 50% windows. For third degree we expect 75% windows to be close to p_0, and 25% windows to be close to p_1. For unrelated, we expect the proportion of differences to stay close to p_0 with some noise. (A) AITI95-AITI98 (READ estimates unrelated, lcMLkin and KIN predict second degree. (B) AITI72-AITI77A (READ estimates unrelated, lcMLkin shows unrelated, and KIN predicts third degree). (C) POST131-POST28 (READ estimates Unrelated, lcMLkin shows 3rd-5th degree, and KIN predicts second degree.) (D) ALT3-ALT4 (READ estimates unrelated, lcMLkin shows second degree and KIN predicts third degree.)

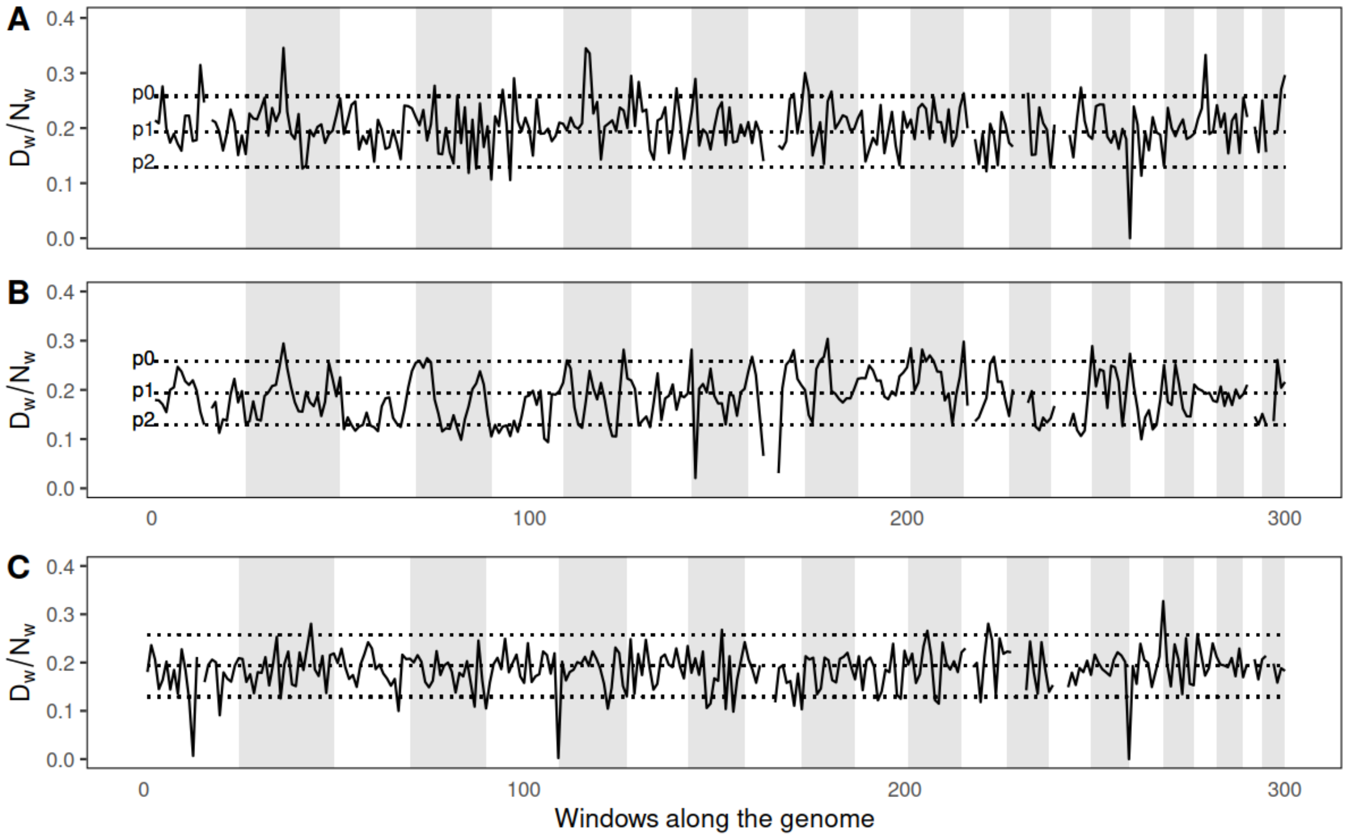

Fig S 12: Plots showing proportion of differences in windows along the genome for the first degree relatives for which KIN differs from lcMLkin. Note that we expect to see proportion of differences for siblings to be close to p_0 in 25% windows, p_1 in 50% windows, and p_2 in 25% windows. For parent-child, we expect the proportion of differences to stay close to p_1 with some noise. (A) AITI43-AITI55 (READ estimates first degree, lcMLkin predicts siblings, while KIN predicts parent-child. (B) AITI70-AITI72 (READ estimates first degree, lcMLkin predicts parent-child, while KIN predicts siblings. (C) OBKR76-POST99 (READ estimates first degree, lcMLkin predicts siblings, while KIN predicts parent-child.

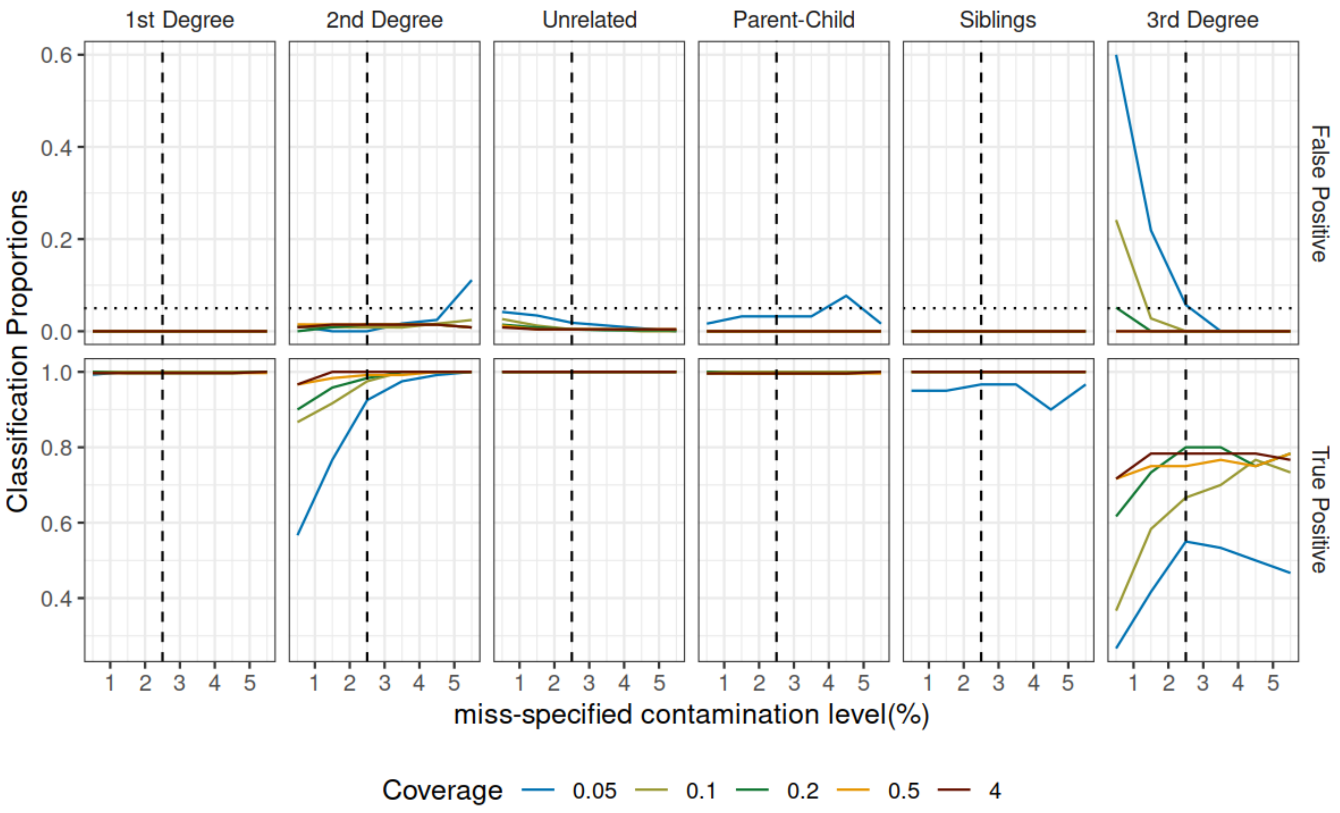

Fig S 13: False positive and true positive rates as a function of incorrect contamination estimates provided to KIN. Dashed vertical line represents true contamination (2.5%) in simulations, and dotted horizontal line in top panel represents 5% false positive rate. For this analysis we varied the estimated contamination level in one individual from 0.5% to 5.5% while keeping contamination estimates for everyone else constant. We then analyzed the relatedness classification for this individual with all other individuals for power analysis.
