# Supplementary material for: KIN: a method to infer relatedness from low-coverage ancient DNA
Source: Genome Biol. 2023 Jan 17;24:10. doi: 10.1186/s13059-023-02847-7 (PMC9843908; doi:10.1186/s13059-023-02847-7)
Supplement: Supplementary file 2 — Additional file 2: Supplementary Tables. Table S1. Relatedness and IBD estimates for Chagyrskaya and Okladnikov cave samples. Table S2. Relatedness estimates for Bronze age samples for which READ and KIN differ, and KIN has log likelihood ratio > 1. Table S3. Relatedness estimates for Bronze age samples for which lcMLkin and KIN differ, and KIN has log likelihood ratio > 1. [file 13059_2023_2847_MOESM2_ESM.docx]

Table S1: Relatedness and IBD estimates for Chagyrskaya and Okladnikov cave samples

| Pair | Relatedness | Second Guess | Log Likelihood Ratio | Within Degree Second Guess | Within Degree Log Likelihood Ratio | k0 | k1 | k2 | IBD Length | IBD Number |
| --- | --- | --- | --- | --- | --- | --- | --- | --- | --- | --- |
| Chagyrskaya01_._Chagyrskaya02 | Unrelated | Third Degree | 1.13 |  |  | 0.875 | 0.125 | 0.0 | 0 | 1 |
| Chagyrskaya01_._Chagyrskaya06 | Unrelated | Third Degree | 5.56 |  |  | 1.0 | 0.0 | 0.0 | 0 | 1 |
| Chagyrskaya01_._Chagyrskaya07 | Unrelated | Third Degree | 2.82 |  |  | 0.936 | 0.064 | 0.0 | 0 | 1 |
| Chagyrskaya01_._Chagyrskaya1141 | Unrelated | Third Degree | 0.7 |  |  | 1.0 | 0.0 | 0.0 | 0 | 1 |
| Chagyrskaya01_._Chagyrskaya12 | Unrelated | Third Degree | 4.4 |  |  | 1.0 | 0.0 | 0.0 | 0 | 1 |
| Chagyrskaya01_._Chagyrskaya13 | Unrelated | Third Degree | 1.64 |  |  | 0.937 | 0.063 | 0.0 | 0 | 1 |
| Chagyrskaya01_._Chagyrskaya14 | Unrelated | Third Degree | 0.36 |  |  | 1.0 | 0.0 | 0.0 | 0 | 1 |
| Chagyrskaya01_._Chagyrskaya17 | Unrelated | Third Degree | 0.86 |  |  | 0.936 | 0.064 | 0.0 | 0 | 1 |
| Chagyrskaya01_._Chagyrskaya18 | Unrelated | Third Degree | 0.11 |  |  | 0.867 | 0.133 | 0.0 | 0 | 1 |
| Chagyrskaya01_._Chagyrskaya19 | Unrelated | Third Degree | 1.82 |  |  | 1.0 | 0.0 | 0.0 | 0 | 1 |
| Chagyrskaya01_._Chagyrskaya20 | Unrelated | Third Degree | 0.89 |  |  | 0.876 | 0.124 | 0.0 | 0 | 1 |
| Chagyrskaya01_._Chagyrskaya41 | Unrelated | Third Degree | 5.15 |  |  | 1.0 | 0.0 | 0.0 | 0 | 1 |
| Chagyrskaya01_._Chagyrskaya60 | Second Degree | Third Degree | 4.05 |  |  | 0.479 | 0.521 | 0.0 | 149 | 8 |
| Chagyrskaya01_._Okladnikov11 | Unrelated | Third Degree | 2.29 |  |  | 1.0 | 0.0 | 0.0 | 0 | 1 |
| Chagyrskaya01_._Okladnikov15 | Unrelated | Third Degree | 2.2 |  |  | 1.0 | 0.0 | 0.0 | 0 | 1 |
| Chagyrskaya02_._Chagyrskaya06 | Unrelated | Third Degree | 5.75 |  |  | 1.0 | 0.0 | 0.0 | 0 | 1 |
| Chagyrskaya02_._Chagyrskaya07 | Unrelated | Third Degree | 2.82 |  |  | 0.915 | 0.085 | 0.0 | 6 | 3 |
| Chagyrskaya02_._Chagyrskaya1141 | Unrelated | Third Degree | 1.24 |  |  | 1.0 | 0.0 | 0.0 | 0 | 1 |
| Chagyrskaya02_._Chagyrskaya12 | Unrelated | Third Degree | 2.37 |  |  | 0.928 | 0.072 | 0.0 | 0 | 1 |
| Chagyrskaya02_._Chagyrskaya13 | Unrelated | Third Degree | 1.77 |  |  | 0.877 | 0.123 | 0.0 | 10 | 1 |
| Chagyrskaya02_._Chagyrskaya14 | Unrelated | Third Degree | 1.9 |  |  | 1.0 | 0.0 | 0.0 | 0 | 1 |
| Chagyrskaya02_._Chagyrskaya17 | Unrelated | Third Degree | 1.82 |  |  | 0.928 | 0.072 | 0.0 | 0 | 1 |
| Chagyrskaya02_._Chagyrskaya18 | Unrelated | Third Degree | 1.52 |  |  | 0.879 | 0.121 | 0.0 | 0 | 1 |
| Chagyrskaya02_._Chagyrskaya19 | Unrelated | Third Degree | 0.35 |  |  | 0.856 | 0.144 | 0.0 | 0 | 1 |
| Chagyrskaya02_._Chagyrskaya20 | Unrelated | Third Degree | 0.06 |  |  | 0.837 | 0.163 | 0.0 | 16 | 2 |
| Chagyrskaya02_._Chagyrskaya41 | Unrelated | Third Degree | 3.17 |  |  | 0.936 | 0.064 | 0.0 | 0 | 1 |
| Chagyrskaya02_._Chagyrskaya60 | Unrelated | Third Degree | 1.42 |  |  | 0.854 | 0.146 | 0.0 | 19 | 4 |
| Chagyrskaya02_._Okladnikov11 | Unrelated | Third Degree | 2.94 |  |  | 1.0 | 0.0 | 0.0 | 0 | 1 |
| Chagyrskaya02_._Okladnikov15 | Unrelated | Third Degree | 2.26 |  |  | 1.0 | 0.0 | 0.0 | 0 | 1 |
| Chagyrskaya06_._Chagyrskaya07 | Unrelated | Third Degree | 3.32 |  |  | 0.939 | 0.061 | 0.0 | 0 | 1 |
| Chagyrskaya06_._Chagyrskaya1141 | Second Degree | Third Degree | 0.12 |  |  | 0.501 | 0.499 | 0.0 | 0 | 1 |
| Chagyrskaya06_._Chagyrskaya12 | Unrelated | Third Degree | 8.26 |  |  | 1.0 | 0.0 | 0.0 | 0 | 1 |
| Chagyrskaya06_._Chagyrskaya13 | Unrelated | Third Degree | 5.34 |  |  | 1.0 | 0.0 | 0.0 | 0 | 1 |
| Chagyrskaya06_._Chagyrskaya14 | Parent-Child | Second Degree | 1.12 | Siblings | 0.35 | 0.0 | 1.0 | 0.0 | 264 | 1 |
| Chagyrskaya06_._Chagyrskaya17 | Unrelated | Third Degree | 2.22 |  |  | 1.0 | 0.0 | 0.0 | 0 | 1 |
| Chagyrskaya06_._Chagyrskaya18 | Unrelated | Third Degree | 3.42 |  |  | 1.0 | 0.0 | 0.0 | 0 | 1 |
| Chagyrskaya06_._Chagyrskaya19 | Unrelated | Third Degree | 4.79 |  |  | 1.0 | 0.0 | 0.0 | 0 | 1 |
| Chagyrskaya06_._Chagyrskaya20 | Unrelated | Third Degree | 5.13 |  |  | 1.0 | 0.0 | 0.0 | 0 | 1 |
| Chagyrskaya06_._Chagyrskaya41 | Unrelated | Third Degree | 6.75 |  |  | 1.0 | 0.0 | 0.0 | 0 | 1 |
| Chagyrskaya06_._Chagyrskaya60 | Unrelated | Third Degree | 7.36 |  |  | 1.0 | 0.0 | 0.0 | 0 | 1 |
| Chagyrskaya06_._Okladnikov11 | Unrelated | Third Degree | 1.21 |  |  | 1.0 | 0.0 | 0.0 | 0 | 1 |
| Chagyrskaya06_._Okladnikov15 | Unrelated | Third Degree | 6.01 |  |  | 1.0 | 0.0 | 0.0 | 0 | 1 |
| Chagyrskaya07_._Chagyrskaya1141 | Unrelated | Third Degree | 0.35 |  |  | 0.937 | 0.063 | 0.0 | 0 | 1 |
| Chagyrskaya07_._Chagyrskaya12 | Unrelated | Third Degree | 7.33 |  |  | 0.946 | 0.054 | 0.0 | 5 | 2 |
| Chagyrskaya07_._Chagyrskaya13 | Unrelated | Third Degree | 4.32 |  |  | 0.938 | 0.062 | 0.0 | 3 | 1 |
| Chagyrskaya07_._Chagyrskaya14 | Unrelated | Third Degree | 0.06 |  |  | 0.873 | 0.127 | 0.0 | 0 | 1 |
| Chagyrskaya07_._Chagyrskaya17 | Parent-Child | Second Degree | 7.33 | Siblings | 5.85 | 0.0 | 1.0 | 0.0 | 286 | 1 |
| Chagyrskaya07_._Chagyrskaya18 | Unrelated | Third Degree | 1.3 |  |  | 0.869 | 0.131 | 0.0 | 7 | 1 |
| Chagyrskaya07_._Chagyrskaya19 | Unrelated | Third Degree | 1.2 |  |  | 0.869 | 0.131 | 0.0 | 5 | 1 |
| Chagyrskaya07_._Chagyrskaya20 | Unrelated | Third Degree | 1.23 |  |  | 0.858 | 0.142 | 0.0 | 17 | 5 |
| Chagyrskaya07_._Chagyrskaya41 | Unrelated | Third Degree | 3.2 |  |  | 0.924 | 0.076 | 0.0 | 7 | 2 |
| Chagyrskaya07_._Chagyrskaya60 | Unrelated | Third Degree | 4.24 |  |  | 0.929 | 0.071 | 0.0 | 7 | 2 |
| Chagyrskaya07_._Okladnikov11 | Unrelated | Third Degree | 5.07 |  |  | 1.0 | 0.0 | 0.0 | 0 | 1 |
| Chagyrskaya07_._Okladnikov15 | Unrelated | Third Degree | 8.24 |  |  | 1.0 | 0.0 | 0.0 | 0 | 1 |
| Chagyrskaya1141_._Chagyrskaya12 | Unrelated | Third Degree | 1.72 |  |  | 1.0 | 0.0 | 0.0 | 0 | 1 |
| Chagyrskaya1141_._Chagyrskaya13 | Identical | Parent-Child | 2.4 |  |  | 0.0 | 0.0 | 1.0 | 0 | 1 |
| Chagyrskaya1141_._Chagyrskaya14 | Unrelated | Third Degree | 0.28 |  |  | 1.0 | 0.0 | 0.0 | 0 | 1 |
| Chagyrskaya1141_._Chagyrskaya17 | Unrelated | Third Degree | 0.85 |  |  | 1.0 | 0.0 | 0.0 | 0 | 1 |
| Chagyrskaya1141_._Chagyrskaya18 | Unrelated | Third Degree | 0.05 |  |  | 0.874 | 0.126 | 0.0 | 0 | 1 |
| Chagyrskaya1141_._Chagyrskaya19 | Identical | Siblings | 5.98 |  |  | 0.0 | 0.0 | 1.0 | 0 | 1 |
| Chagyrskaya1141_._Chagyrskaya20 | Unrelated | Third Degree | 0.52 |  |  | 1.0 | 0.0 | 0.0 | 0 | 1 |
| Chagyrskaya1141_._Chagyrskaya41 | Unrelated | Third Degree | 0.54 |  |  | 1.0 | 0.0 | 0.0 | 0 | 1 |
| Chagyrskaya1141_._Chagyrskaya60 | Unrelated | Third Degree | 0.37 |  |  | 0.937 | 0.063 | 0.0 | 0 | 1 |
| Chagyrskaya1141_._Okladnikov11 | Third Degree | Unrelated | 0.01 |  |  | 0.747 | 0.253 | 0.0 | 0 | 1 |
| Chagyrskaya1141_._Okladnikov15 | Unrelated | Third Degree | 0.27 |  |  | 1.0 | 0.0 | 0.0 | 0 | 1 |
| Chagyrskaya12_._Chagyrskaya13 | Unrelated | Third Degree | 6.45 |  |  | 1.0 | 0.0 | 0.0 | 0 | 1 |
| Chagyrskaya12_._Chagyrskaya14 | Unrelated | Third Degree | 1.45 |  |  | 1.0 | 0.0 | 0.0 | 0 | 1 |
| Chagyrskaya12_._Chagyrskaya17 | Unrelated | Third Degree | 7.21 |  |  | 1.0 | 0.0 | 0.0 | 0 | 1 |
| Chagyrskaya12_._Chagyrskaya18 | Unrelated | Third Degree | 3.24 |  |  | 0.935 | 0.065 | 0.0 | 0 | 1 |
| Chagyrskaya12_._Chagyrskaya19 | Unrelated | Third Degree | 2.11 |  |  | 0.931 | 0.069 | 0.0 | 0 | 1 |
| Chagyrskaya12_._Chagyrskaya20 | Unrelated | Third Degree | 4.66 |  |  | 0.934 | 0.066 | 0.0 | 0 | 1 |
| Chagyrskaya12_._Chagyrskaya41 | Unrelated | Third Degree | 9.55 |  |  | 1.0 | 0.0 | 0.0 | 0 | 1 |
| Chagyrskaya12_._Chagyrskaya60 | Unrelated | Third Degree | 5.48 |  |  | 0.936 | 0.064 | 0.0 | 3 | 2 |
| Chagyrskaya12_._Okladnikov11 | Unrelated | Third Degree | 4.89 |  |  | 1.0 | 0.0 | 0.0 | 0 | 1 |
| Chagyrskaya12_._Okladnikov15 | Unrelated | Third Degree | 6.3 |  |  | 1.0 | 0.0 | 0.0 | 0 | 1 |
| Chagyrskaya13_._Chagyrskaya14 | Unrelated | Third Degree | 0.3 |  |  | 1.0 | 0.0 | 0.0 | 0 | 1 |
| Chagyrskaya13_._Chagyrskaya17 | Third Degree | Unrelated | 0.66 |  |  | 0.747 | 0.253 | 0.0 | 27 | 2 |
| Chagyrskaya13_._Chagyrskaya18 | Unrelated | Third Degree | 3.33 |  |  | 1.0 | 0.0 | 0.0 | 0 | 1 |
| Chagyrskaya13_._Chagyrskaya19 | Identical | Siblings | 40.17 |  |  | 0.0 | 0.0 | 1.0 | 0 | 1 |
| Chagyrskaya13_._Chagyrskaya20 | Unrelated | Third Degree | 4.13 |  |  | 0.937 | 0.063 | 0.0 | 0 | 1 |
| Chagyrskaya13_._Chagyrskaya41 | Unrelated | Third Degree | 1.1 |  |  | 0.87 | 0.13 | 0.0 | 0 | 1 |
| Chagyrskaya13_._Chagyrskaya60 | Third Degree | Unrelated | 0.2 |  |  | 0.783 | 0.217 | 0.0 | 27 | 2 |
| Chagyrskaya13_._Okladnikov11 | Unrelated | Third Degree | 1.58 |  |  | 1.0 | 0.0 | 0.0 | 0 | 1 |
| Chagyrskaya13_._Okladnikov15 | Unrelated | Third Degree | 5.16 |  |  | 1.0 | 0.0 | 0.0 | 0 | 1 |
| Chagyrskaya14_._Chagyrskaya17 | Unrelated | Third Degree | 0.22 |  |  | 1.0 | 0.0 | 0.0 | 0 | 1 |
| Chagyrskaya14_._Chagyrskaya18 | Unrelated | Third Degree | 0.86 |  |  | 1.0 | 0.0 | 0.0 | 0 | 1 |
| Chagyrskaya14_._Chagyrskaya19 | Second Degree | Third Degree | 0.01 |  |  | 0.498 | 0.502 | 0.0 | 62 | 1 |
| Chagyrskaya14_._Chagyrskaya20 | Unrelated | Third Degree | 0.01 |  |  | 0.872 | 0.128 | 0.0 | 0 | 1 |
| Chagyrskaya14_._Chagyrskaya41 | Unrelated | Third Degree | 0.16 |  |  | 0.936 | 0.064 | 0.0 | 0 | 1 |
| Chagyrskaya14_._Chagyrskaya60 | Unrelated | Third Degree | 0.9 |  |  | 1.0 | 0.0 | 0.0 | 0 | 1 |
| Chagyrskaya14_._Okladnikov11 | Unrelated | Third Degree | 0.57 |  |  | 1.0 | 0.0 | 0.0 | 0 | 1 |
| Chagyrskaya14_._Okladnikov15 | Unrelated | Third Degree | 0.38 |  |  | 1.0 | 0.0 | 0.0 | 0 | 1 |
| Chagyrskaya17_._Chagyrskaya18 | Unrelated | Third Degree | 0.93 |  |  | 0.875 | 0.125 | 0.0 | 0 | 1 |
| Chagyrskaya17_._Chagyrskaya19 | Third Degree | Unrelated | 0.76 |  |  | 0.748 | 0.252 | 0.0 | 9 | 1 |
| Chagyrskaya17_._Chagyrskaya20 | Unrelated | Third Degree | 1.48 |  |  | 0.881 | 0.119 | 0.0 | 0 | 1 |
| Chagyrskaya17_._Chagyrskaya41 | Third Degree | Unrelated | 0.19 |  |  | 0.757 | 0.243 | 0.0 | 0 | 1 |
| Chagyrskaya17_._Chagyrskaya60 | Third Degree | Unrelated | 2.05 |  |  | 0.726 | 0.274 | 0.0 | 45 | 5 |
| Chagyrskaya17_._Okladnikov11 | Unrelated | Third Degree | 0.76 |  |  | 1.0 | 0.0 | 0.0 | 0 | 1 |
| Chagyrskaya17_._Okladnikov15 | Unrelated | Third Degree | 4.86 |  |  | 1.0 | 0.0 | 0.0 | 0 | 1 |
| Chagyrskaya18_._Chagyrskaya19 | Unrelated | Third Degree | 0.77 |  |  | 0.872 | 0.128 | 0.0 | 0 | 1 |
| Chagyrskaya18_._Chagyrskaya20 | Unrelated | Third Degree | 2.48 |  |  | 0.932 | 0.068 | 0.0 | 0 | 1 |
| Chagyrskaya18_._Chagyrskaya41 | Unrelated | Third Degree | 1.46 |  |  | 0.871 | 0.129 | 0.0 | 0 | 1 |
| Chagyrskaya18_._Chagyrskaya60 | Unrelated | Third Degree | 1.93 |  |  | 0.876 | 0.124 | 0.0 | 6 | 1 |
| Chagyrskaya18_._Okladnikov11 | Unrelated | Third Degree | 2.89 |  |  | 1.0 | 0.0 | 0.0 | 0 | 1 |
| Chagyrskaya18_._Okladnikov15 | Unrelated | Third Degree | 4.06 |  |  | 1.0 | 0.0 | 0.0 | 0 | 1 |
| Chagyrskaya19_._Chagyrskaya20 | Unrelated | Third Degree | 1.59 |  |  | 0.871 | 0.129 | 0.0 | 0 | 1 |
| Chagyrskaya19_._Chagyrskaya41 | Third Degree | Second Degree | 0.07 |  |  | 0.724 | 0.276 | 0.0 | 20 | 2 |
| Chagyrskaya19_._Chagyrskaya60 | Third Degree | Unrelated | 0.25 |  |  | 0.765 | 0.235 | 0.0 | 28 | 3 |
| Chagyrskaya19_._Okladnikov11 | Unrelated | Third Degree | 0.6 |  |  | 1.0 | 0.0 | 0.0 | 0 | 1 |
| Chagyrskaya19_._Okladnikov15 | Unrelated | Third Degree | 3.97 |  |  | 1.0 | 0.0 | 0.0 | 0 | 1 |
| Chagyrskaya20_._Chagyrskaya41 | Unrelated | Third Degree | 3.33 |  |  | 0.937 | 0.063 | 0.0 | 0 | 1 |
| Chagyrskaya20_._Chagyrskaya60 | Unrelated | Third Degree | 3.7 |  |  | 0.931 | 0.069 | 0.0 | 4 | 1 |
| Chagyrskaya20_._Okladnikov11 | Unrelated | Third Degree | 3.87 |  |  | 1.0 | 0.0 | 0.0 | 0 | 1 |
| Chagyrskaya20_._Okladnikov15 | Unrelated | Third Degree | 1.35 |  |  | 0.935 | 0.065 | 0.0 | 0 | 1 |
| Chagyrskaya41_._Chagyrskaya60 | Unrelated | Third Degree | 1.27 |  |  | 0.872 | 0.128 | 0.0 | 0 | 1 |
| Chagyrskaya41_._Okladnikov11 | Unrelated | Third Degree | 4.62 |  |  | 1.0 | 0.0 | 0.0 | 0 | 1 |
| Chagyrskaya41_._Okladnikov15 | Unrelated | Third Degree | 6.58 |  |  | 1.0 | 0.0 | 0.0 | 0 | 1 |
| Chagyrskaya60_._Okladnikov11 | Unrelated | Third Degree | 3.28 |  |  | 1.0 | 0.0 | 0.0 | 0 | 1 |
| Chagyrskaya60_._Okladnikov15 | Unrelated | Third Degree | 6.61 |  |  | 1.0 | 0.0 | 0.0 | 0 | 1 |
| Okladnikov11_._Okladnikov15 | Unrelated | Third Degree | 1.47 |  |  | 1.0 | 0.0 | 0.0 | 0 | 1 |

Table S2: Relatedness estimates for Bronze age samples for which READ and KIN differ, and KIN has log likelihood ratio > 1

| Relatedness KIN | Log liklihood ratio (KIN) | Relatedness READ | Z_upper (READ) | Z_lower (READ) | Relatedness lcMLkin | pair | nSNPs |
| --- | --- | --- | --- | --- | --- | --- | --- |
| Third Degree | 2.84 | Unrelated |  | -3.31 | Third Degree | AITI119_AITI2 | 454424 |
| Third Degree | 1.39 | Unrelated |  | -1.66 | Third Degree | AITI119_AITI55 | 25914 |
| Third Degree | 1.98 | Unrelated |  | -4.02 | Third Degree | AITI120_AITI2 | 450125 |
| Third Degree | 2.05 | Unrelated |  | -1.16 | Third Degree | AITI120_AITI55 | 27306 |
| Second Degree | 2.53 | Unrelated |  | -0.89 | Second Degree | AITI2_AITI55 | 33929 |
| Third Degree | 2.84 | Unrelated |  | -2.05 | Third Degree | AITI2_AITI72 | 587530 |
| Parent-offspring | 1.66 | Second Degree | 3.1 | -0.78 | too low coverage | AITI33_OTTM91 | 5898 |
| Third Degree | 1.64 | Unrelated |  | -0.46 | Third Degree | AITI36_AITI72 | 18868 |
| Third Degree | 1.31 | Unrelated |  | -2.21 | too low coverage | AITI40_AITI62B | 8275 |
| Third Degree | 3.8 | Unrelated |  | -2.41 | Third Degree | AITI40_AITI72 | 174552 |
| Third Degree | 2.27 | Unrelated |  | -1.78 | Third Degree | AITI40_AITI77A | 80217 |
| Parent-offspring | 7.35 | IdenticalTwins/SameIndividual | 0.04 |  | too low coverage | AITI62B_OTTM156 | 8024 |
| Third Degree | 1.37 | Unrelated |  | -8.72 | Unrelated | AITI72_AITI77A | 224138 |
| Third Degree | 1.79 | Unrelated |  | -8.3 | Third Degree | AITI72_AITI77B | 260000 |
| Parent-offspring | 7.07 | Second Degree | 5.3 | -0.1 | too low coverage | AITI86_AITI87 | 12479 |
| Second Degree | 1.95 | Unrelated |  | -1.11 | Second Degree | AITI95_AITI98 | 249170 |
| Third Degree | 1.2 | Unrelated |  | -0.38 | Second Degree | ALT3_ALT4 | 35020 |
| Third Degree | 1.23 | Unrelated |  | -2.34 | too low coverage | OBKR66_OBKR82 | 7608 |
| Third Degree | 2.54 | Unrelated |  | -6.06 | Third Degree | OBKR67_OBKR80 | 280504 |
| Third Degree | 2.79 | Unrelated |  | -5.61 | Third Degree | OBKR86_WEHR1586 | 446568 |
| Third Degree | 1.78 | Unrelated |  | -4.23 | Third Degree | POST1_POST50 | 40875 |
| Second Degree | 1.81 | Unrelated |  | -1.3 | Third Degree | POST131_POST28 | 28501 |
| Third Degree | 1.62 | Unrelated |  | -1.9 | too low coverage | POST140_POST85 | 8784 |
| Third Degree | 1.05 | Unrelated |  | -2.86 | Third Degree | POST35_POST85 | 19786 |
| Third Degree | 1.17 | Unrelated |  | -1.78 | Third Degree | POST50_POST85 | 18548 |
| Third Degree | 2.19 | Unrelated |  | -1.17 | too low coverage | UNTA58147_UNTA58152 | 15001 |
| Parent-offspring | 8.77 | Second Degree | 4.54 | -0.06 | too low coverage | UNTA5867_UNTA5868Sk1 | 8614 |
| Parent-offspring | 2.52 | Second Degree | 4.7 | -0.48 | too low coverage | UNTA5867_UNTA5868Sk2 | 10554 |

Table S3: Relatedness estimates for Bronze age samples for which lcMLkin and KIN differ, and KIN has log likelihood ratio > 1

| Relatedness KIN | Log liklihood ratio (KIN) | Relatedness READ | Z_upper (READ) | Z_lower (READ) | Relatedness lcMLkin | pair | nSNPs |
| --- | --- | --- | --- | --- | --- | --- | --- |
| Unrelated | 4.17 | Unrelated |  | -4.62 | Third Degree | AITI37_AITI77B | 22980 |
| Unrelated | 8.29 | Unrelated |  | -6.05 | Third Degree | AITI37_HUGO169Sk1 | 36775 |
| Parent-offspring | 15.0 | First Degree | 0.59 | -12.48 | Sibling | AITI43_AITI55 | 35981 |
| Unrelated | 1.55 | Unrelated |  | -3.98 | Third Degree | AITI43_AITI86 | 35416 |
| Unrelated | 2.49 | Unrelated |  | -8.95 | Third Degree | AITI66_OBKR66 | 293409 |
| Sibling | 71.98 | First Degree | 15.91 | -7.53 | Parent-offspring | AITI70_AITI72 | 332171 |
| Third Degree | 1.37 | Unrelated |  | -8.72 | Unrelated | AITI72_AITI77A | 224138 |
| Third Degree | 1.2 | Unrelated |  | -0.38 | Second Degree | ALT3_ALT4 | 35020 |
| Unrelated | 9.78 | Unrelated |  | -7.41 | Third Degree | HUGO180Sk1_UNTA851412 | 240420 |
| Unrelated | 1.25 | Unrelated |  | -7.27 | Third Degree | OBKR2_OBKR93 | 102308 |
| Parent-offspring | 32.67 | First Degree | 4.73 | -6.81 | Sibling | OBKR76_POST99 | 23773 |
| Unrelated | 8.65 | Unrelated |  | -4.85 | Third Degree | OBKR96_POST50 | 87341 |
| Unrelated | 3.22 | Unrelated |  | -3.11 | Third Degree | OTTM151ind1_OTTM81 | 18098 |
| Second Degree | 1.81 | Unrelated |  | -1.3 | Third Degree | POST131_POST28 | 28501 |
| Unrelated | 1.58 | Unrelated |  | -8.32 | Third Degree | POST140_POST44 | 286726 |
| Unrelated | 3.34 | Unrelated |  | -5.39 | Third Degree | WEHR1414_WEHR1474 | 106303 |
