# Supplementary material for: KIN: a method to infer relatedness from low-coverage ancient DNA
Source: Genome Biol. 2023 Jan 17;24:10. doi: 10.1186/s13059-023-02847-7 (PMC9843908; doi:10.1186/s13059-023-02847-7)
Supplement: Supplementary file 3 — Additional file 3. Review history. [file 13059_2023_2847_MOESM3_ESM.docx]

Review History

**First round of review**

**Reviewer 1**

**Were you able to assess all statistics in the manuscript, including the appropriateness of statistical tests used?** Yes, and I have assessed the statistics in my report.

**Were you able to directly test the methods?** No

**Comments to author:**

Popli et al. developed KIN, a method for relatedness inference in low coverage ancient DNA (aDNA). Relatedness inference is an important problem, with many methods applicable to modern DNA, but comparatively fewer that apply to aDNA. Understanding the cultures, burial practices, etc. of ancient populations is enhanced by reliable detection of relatives, and this study adds to the suite of available methods for this inference. Importantly, KIN accounts for contamination and ascertainment bias so prevalent in aDNA. As well, KIN's ability to detect runs of homozygosity (ROH) can be important as inbreeding is common in an aDNA context and ROHs can bias relatedness inference.

My biggest concern with the present manuscript is the model used for simulating relatives and the fact that this model appears to be the same one used for inference. Cabellero et al. (2019) showed that for close relatives, sex-specific genetic maps and crossover interference affect IBD segment distributions. Might it be worth at least simulating data using these models? Using the same model for simulation and inference is never ideal, and, in this case, a more realistic simulation models exist and could be applied. (Since interference reduces the variance in relatedness, KIN's performance might even improve after doing this.)

Another question is regarding Figure 4: Is it expected that the accuracy (is this precision?) would be lower for siblings vs 2nd degree relatives and lower for 2nd degree vs 3rd degree? This seems counter-intuitive and I may not be reading the plot right.

Line 420: Something that would be helpful to discuss is how well various methods work for estimating contamination. Similarly, how robust is KIN to misspecification?

Minor comments:

This probably has limited impact on the results, but is it worth setting \pi, the initial probabilities, to the expected rates of each hidden state? Perhaps the information may be abundant enough, even in a low coverage context, that the data overwhelms the priors.

Lines 180-1: "We use this equation [(4)] even if preprocessing steps result in non-integer D_w and N_w …" I may have miss it, but it might be nice to say here what leads to such non-integer values.

Line 236: "the number of sites with at least two reads M." Is M a vector? It's not subscripted in Equation (7).

Line 243-4: This is a little nitpicky, but since you're using Y for the hidden states for the ROH HMM (and Z for the relatedness one), should the p_i's also use a different notation? q_i?

Line 260: Again pedantic, but should this say C_ij = C_i + C_j, i.e., not approximately equal, since this seems to be the definition of C_ij?

In Equations (9) and below, it may be good to emphasize that D here is an event, not a number. It's clear enough with a close read, but I found it somewhat confusing in light of D_w earlier.

Line 297: "Surprisingly, the bias was lower for lower coverages." - Does this suggest that one should down sample? Alternatively is there a higher coverage level that avoids this bias? Perhaps there's a part of the coverage space that's upwardly biased?

Lines 366-8: Just to confirm: the k0 and r numbers given on line 368 are from lcMLkin?

Lines 374-6: What about cases where READ is confident and its classifications differ from KIN?

Lines 379-80: "lcMLkin infers a group of relatedness together as 3rd - 5th, it is likely that KIN is able to correctly resolve these cases." I wonder if this is overstated given the results in Figure 5?

Line 382: "likely examples of 4th or 5th degree relations" - might they be more distant, too?

Lines 383-9: It might be helpful to reason about what the truth is for the discrepant cases (or say that the results are unclear).

Lines 429-30: "as parent-child, which is in agreement with the finding that the mtDNA haplotypes differ between the samples" - so is one of the samples male?

Line 436-7: "KIN may show inaccurate inference of p0 causing inaccurate relatedness inference" not important, but if it's easy to add, could the authors explain which direction the bias would tend to? Does it depends on the form the structure takes?

Typos:

- Line 60: "incproporate"

- A little below line 301: no closing parenthesis: "or contamination, …"

- Line 304: "relatdness"

- Line 343: "atleast"

- Line 373: should the delta LL be > 1 (not <)?

- Line 409: "upto"

- Line 451: "bamfiles"

**Reviewer 2**

**Were you able to assess all statistics in the manuscript, including the appropriateness of statistical tests used?** Yes, and I have assessed the statistics in my report.

**Were you able to directly test the methods?** No

**Comments to author:**

Popli and co-authors describe a program called KIN for inferring relatedness from DNA sequence data. This program covers an important gap in that it can make inferences from small amounts of data and handles contamination. It also models runs-of-homozygosity, which appear to be common in ancient DNA datasets and can distinguish between parental and full-sib (both 1st degree relatives).

The method is well conceived and implemented and the paper is an adequate description of the work. My only issues can be considered minor. They are offered as potentially useful guidance for improving the manuscript.

Comments, questions, and suggestions:

1. The authors state the KIN has no "requirement for a reference panel". It appears, however, that it simply uses the input panel as the "reference" panel for measuring genetic differences between unrelated segments of the genome. This use of a reference panel is distinct from how other programs use it, i.e., to learn allele frequencies at each site. Specifically, the paragraph beginning on line 154 describes how p0 is calculated: by observing the median differences for all possible pairs in the input set, i.e., panel. Perhaps the authors can conjure a phrase that describes the necessary inputs of KIN more exactly.

2. Figure 1 is large and unclear. The colors of the top panel are not labeled or described in the figure legend. The figure could be improved by adding a schematic of the parental chromosomes.

3. In general, the paper reads as though it were born of a technical note describing the method and has not evolved far from that. This style may suit many readers. But I found it to be overly reliant on mathematical descriptions. Many readers may appreciate more verbiage describing the rationale for the equations. A well-crafted descriptive sentence or two introducing an equation is an invitation to understand it. A lone equation, with only a character list of variables is a warning to stay away.

4. In section 2.2, Dw is variously described as "the expected number of pariwise differences", "the number of sites that are different" (i.e., observed differences), and a number that "needs to be estimated". From later context, it's clear that Dw is a number that is observed from analysis of the data. Please don't make the reader work so hard to understand! Clean up this section.

5. Concerning the equation around line 164, introducing p(Zw,Hw), should there not be a term for p4, i.e., the case where the two samples are from the same individual and that individual happens to be in a ROH?

6. Setting p3 to p2/2 (line 167) is noted with unapologetic arbitrariness. Is it that p3 is expected to be half of p2 and p2 is expected to be estimated more accurately than p3 can be? If so - or something like that - please encourage and assist the interested reader.

7. The beta binomial model appears to be appropriate and useful. The motivation for it is that "genetic linkage" increases correlation of site behavior in linked windows. I have my doubts about this. It does not really matter. Whatever the cause of the increased variance, it is handled. However, the authors could devise an experiment (same data, randomized site location, e.g.) to determine if the increased variance is attributable to linkage.

8. Section 2.6, "Because the models we test are not nested…" - it's not clear what "nested" means in this context. Does "nested" mean "independent" or "non-overlapping" or something like that. Perhaps nested models is a common term describing model relationships. I, however, am not familiar with it and would benefit from a less jargony description.

9. Figure 2: I fail to understand why the third panel, "Identical", does not show increased likelihood in sections where the sibs co-inherit the same chromosome from both parents. Similarly, in the parent-child model, there should be a likelihood bump when the siblings have one chromosome identical by descent.

10. Figure 2: This figure needs a title. That title should have the phrase "simulated siblings" in it. As it is, the reader needs to get to the third line of the caption to learn what the figure is about.

11. ROH detection section - the accuracy of the ROH HMM is reported in "bias" and "variance". These metrics are non-intuitive. Why not report the accuracy in terms of sensitivity & specificity at some reasonable cutoff?

12. Line 327, as written, it's not clear if the "(<3%)" describes the level of contamination or the true positive rate.

13. The authors chose to report their relatedness result as the LLR between the two best models. As they point out, there are discrete models of relatedness. For example, one can be 50% or 100% IBD, but not 75%. As relatedness becomes more distant, the distance between them decreases. Thus, under this framework, it is necessarily more difficult to distinguish between competing models of distant relatedness than it is between competing models of more close relatives. That is, even with perfect data, the LLR comparing 4th versus 5th degree relatives will be lower than the LLR between 1st and 2nd degree relatives. For this reason, it may be possible to derive a maximum likelihood or other classifier that takes the likelihoods of all models.

**Authors Response**

**Point-by-point responses to the reviewers’ comments:**

Reviewer #1: Popli et al. developed KIN, a method for relatedness inference in low coverage ancient DNA (aDNA). Relatedness inference is an important problem, with many methods applicable to modern DNA, but comparatively fewer that apply to aDNA. Understanding the cultures, burial practices, etc. of ancient populations is enhanced by reliable detection of relatives, and this study adds to the suite of available methods for this inference. Importantly, KIN accounts for contamination and ascertainment bias so prevalent in aDNA. As well, KIN's ability to detect runs of homozygosity (ROH) can be important as inbreeding is common in an aDNA context and ROHs can bias relatedness inference.

My biggest concern with the present manuscript is the model used for simulating relatives and the fact that this model appears to be the same one used for inference. Cabellero et al. (2019) showed that for close relatives, sex-specific genetic maps and crossover interference affect IBD segment distributions. Might it be worth at least simulating data using these models? Using the same model for simulation and inference is never ideal, and, in this case, a more realistic simulation models exist and could be applied. (Since interference reduces the variance in relatedness, KIN's performance might even improve after doing this.)

We have now edited the testing simulations, and use pedsim (Cabellero et al., 2019) to simulate IBD sharing for close relatives. We describe this in lines 477-83: There are two different ways in which we generate recombination points. For the estimation of transition probabilities, we simulate recombination by first drawing the number of breakpoints as a Poisson random variable with parameter rL, and use a uniform distribution on [1, L] to sample the positions of recombination points. For the testing of our method, we use pedsim (Caballero et al. 2019) to simulate

recombination points. This allows us to take into account sex-specific recombination rates and crossover interference, and thus is expected to give a more realistic recombination landscape.

Another question is regarding Figure 4: Is it expected that the accuracy (is this precision?) would be lower for siblings vs 2nd degree relatives and lower for 2nd degree vs 3rd degree? This seems counter-intuitive and I may not be reading the plot right.

Figure 4 indeed shows the accuracy of the IBD predictions, we have made it clearer in the legend of the figure: Accuracy is defined as the proportion of correctly predicted IBD states, when compared to the central position of the window.

The reason that the accuracy of IBD prediction is lowest for siblings, followed by 2^nd^ degree and 3rd degree relatives is because IBD in siblings is more variable, and therefore harder to predict. A pair of 3rd-degree relatives, for example, are expected to only share 12.5% of their genome IBD, with the rest being unrelated. Therefore, even a naive classifier that classified everything as unrelated would have an accuracy of 87.5%. In contrast, siblings are expected to share one chromosome IBD for half their genomes, and both chromosomes for another 25%. This higher variability makes predictions harder.

Line 420: Something that would be helpful to discuss is how well various methods work for estimating contamination. Similarly, how robust is KIN to misspecification?

We now present an analysis where we misspecify the contamination rate and evaluate its effect on KIN’s inference of relatedness. This is now described in the supplementary figure S13 and mentioned on lines 326-31 in the results: In comparison, we also ran KIN, giving it the simulated contamination amounts for each individual. We found that the correction implemented in KIN is sufficient to remove this bias, and the true and false positive rates remain as in the control (Figure 5). We also perform an analysis where we misspecify the contamination, and find that the effect of modest misspecification is small (Fig. S13).

We also discuss the implications in the discussion: 427-30: However, in many cases contamination estimates may be uncertain or inaccurate, and we show in fig. S13 that KIN's performance is robust to small deviations (small compared to average pairwise heterozygosity) in contamination estimates.

We agree that different methods may estimate contamination with various biases, but feel that for our purposes, showing that KIN is reasonably robust to small misestimation of contamination is sufficient, and we feel that a systematic evaluation of different contamination estimation methods is beyond the scope of our paper.

Minor comments:

This probably has limited impact on the results, but is it worth setting \pi, the initial probabilities, to the expected rates of each hidden state? Perhaps the information may be abundant enough, even in a low coverage context, that the data overwhelms the priors.

We agree that this is a good idea, and we actually had implemented it but did not update our manuscript. We now updated the description of the algorithm in line 126-8: The initial probabilities pi give the probabilities of being in each state Z_0 (at the beginning of each chromosome), which we set to the stationary distribution of the transition matrix for simplicity.

Lines 180-1: "We use this equation [(4)] even if preprocessing steps result in non-integer D_w and N_w ..." I may have miss it, but it might be nice to say here what leads to such non-integer values.

We get non-integer values because the contamination correction leads to non-integer values for both the estimated number of differences and the estimated total number of overlapping sites

involving endogenous sequences (N_w). We have made this clearer now in line 167-9 by adding references to relevant sections : We use this equation even if preprocessing steps (see equation 1 and section 2.8) result in non-integer D_w and N_w, in which case we approximate the binomial coefficient using Gamma functions.

Line 236: "the number of sites with at least two reads M." Is M a vector? It's not subscripted in Equation (7).

M is indeed a vector, and we have added the missing subscript in equation 7.

Line 243-4: This is a little nitpicky, but since you're using Y for the hidden states for the ROH HMM (and Z for the relatedness one), should the p_i's also use a different notation? q_i?

We do not use a new notation for the p_2 and p_3 in section 2.7 describing the ROH HMM to make clear that we use the same p_i’s, along with p_0 and p_1, for the relatedness HMM described in section 2.4.

Line 260: Again pedantic, but should this say C_ij = C_i + C_j, i.e., not approximately equal, since this seems to be the definition of C_ij?

We have now changed ‘approximately equal sign’ to equal in line 253.

In Equations (9) and below, it may be good to emphasize that D here is an event, not a number. It's clear enough with a close read, but I found it somewhat confusing in light of D_w earlier.

We now clarified this on line 260: “For any particular comparison showing a difference D (not to be confused with the number of differences D_w), we calculate the probability of the event E that it is between endogenous reads as...”

Line 297: "Surprisingly, the bias was lower for lower coverages." - Does this suggest that one should down sample? Alternatively is there a higher coverage level that avoids this bias? Perhaps there's a part of the coverage space that's upwardly biased?

We agree that this analysis was counter-intuitive. Following the suggestion by reviewer 2 (see also response to point 11), we now present specificity and sensitivity for this analysis. We show the results of our new analysis in table 2 and explain it in lines 287-95: A systematic evaluation of the performance of the ROH-HMM is given in table 2. For the purpose of this analysis, we classified all windows that were at least 20% homozygous as ROH, and the remainder as non-ROH. Likewise, we classified all windows with a posterior probability of ROH of at least 20% as ROH. We used these cases to compute sensitivity (Se) and specificity (Sp). In the control case (simulations with no ascertainment, contamination, or ROH) we see that the specificity remains above 0.99 for all coverages. In the cases where we simulate ROH, we find that sensitivity decreases from 97% at 4x coverage to less than 65% at 0.05x coverage, but specificity remains high at above 0.96 for all cases, suggesting that the number of erroneously called ROH segments is low.

Lines 366-8: Just to confirm: the k0 and r numbers given on line 368 are from lcMLkin?

Yes, the numbers k0 ~ 0.3 and r ~ 0.6 are estimates from lcMLkin as shown in supplementary plot S 10. We also clarify this in the main text by defining the lcMLkin parameters (lines 364-370): We find that lcMLkin creates four different clusters based on the coefficient of relatedness (r) and the proportion of the genome in the unrelated state (k_0) for different relatedness cases (identical, parent-child, 2nd degree and unrelated). However, the location of these clusters strongly deviate from the expected values, likely due to the low coverage and ROH. Remains from the same individual, for example, are expected to be at k_0 = 0 and r = 1, but are at k_0 ~ 0.3, r ~ 0.6 for lcMLkin (Figure S10).

Lines 374-6: What about cases where READ is confident and its classifications differ from KIN?

We reworded this section to be more explicit (lines 377-9): Twenty of these comparisons, KIN predicts to be third-degree relationships, and READ concordantly classifies them as unrelated (as it only infers first and second degree relationships). And later on (lines 382-7): There are a total of 83 pairs where READ obtains a confident call (s.d. > 1), but KIN does not (∆LL < 1). In 80 of these cases, READ classifies them as unrelated, KIN classifies 3rd degree, while lcMLkin predicts 3rd - 5th degree. The remaining cases READ classifies unrelated, while KIN and lcMLkin call a 2nd-degree relationship. Thus, the vast majority of differences can be explained by READ not considering 3rd-degree relationships.

Lines 379-80: "lcMLkin infers a group of relatedness together as 3rd - 5th, it is likely that KIN is able to correctly resolve these cases." I wonder if this is overstated given the results in Figure 5? Line 382: "likely examples of 4th or 5th degree relations" - might they be more distant, too?

We agree that these two statements are speculative and have thus removed them.

Lines 383-9: It might be helpful to reason about what the truth is for the discrepant cases (or say that the results are unclear).

We have edited line 390-1: In the last case, all three methods differ (table S3, Fig. S11), and the true relatedness is unresolved in this case.

Lines 429-30: "as parent-child, which is in agreement with the finding that the mtDNA haplotypes differ between the samples" - so is one of the samples male?

Yes, the authors (Skov et al., 2022, submitted) report that Chagyrskaya07 is male, while Chagyrskaya17 is female. We have added this to main test in lines 436-9:

We show that when applied to Neandertal specimens from Chagyrskaya and Okladnikov Caves, KIN identifies a pair of 1st-Degree relatives as parent-child, which is in agreement with the finding that the mtDNA haplotypes differ between the samples (one sample is male and the other female) (Skov et al., 2022 (accepted)).

Line 436-7: "KIN may show inaccurate inference of p0 causing inaccurate relatedness inference" not important, but if it's easy to add, could the authors explain which direction the bias would tend to? Does it depends on the form the structure takes?

It indeed depends on the structure. If a population is structured into sub populations such that the pairwise heterozygosities among subpopulations are similar to pairwise heterozygosities between subpopulations, then estimation of p_0 would not be affected. However, if average pairwise heterozygosities between subpopulations are much higher than within populations heteozygosities, then p_0 might be overestimated. Also, if average pairwise heterozygosities differ in each subpopulation due to different effective population sizes, then depending on the sample sizes of the subpopulations, the median pairwise heterozygosity p_0 could be overestimated (when subpopulation with high pairwise heterozygosity has higher sample size) or underestimated (when subpopulation with low pairwise heterozygosity has higher sample size).

Because these issues are quite subtle and depend on the details of the population structure, we chose not to directly address them in the paper.

Typos:

- Line 60: "incproporate"

- A little below line 301: no closing parenthesis: "or contamination, ..."

- Line 304: "relatdness"

- Line 343: "atleast"

- Line 373: should the delta LL be > 1 (not <)?

- Line 409: "upto"

- Line 451: "bamfiles"

We have now corrected these typos.

Reviewer #2: Popli and co-authors describe a program called KIN for inferring relatedness from DNA sequence data. This program covers an important gap in that it can make inferences from small amounts of data and handles contamination. It also models runs-of-homozygosity, which appear to be common in ancient DNA datasets and can distinguish between parental and full-sib (both 1st degree relatives).

The method is well conceived and implemented and the paper is an adequate description of the work. My only issues can be considered minor. They are offered as potentially useful guidance for improving the manuscript.

Comments, questions, and suggestions:

1. The authors state the KIN has no "requirement for a reference panel". It appears, however, that it simply uses the input panel as the "reference" panel for measuring genetic differences between unrelated segments of the genome. This use of a reference panel is distinct from how other programs use it, i.e., to learn allele frequencies at each site. Specifically, the paragraph beginning on line 154 describes how p0 is calculated: by observing the median differences for all possible pairs in the input set, i.e., panel. Perhaps the authors can conjure a phrase that describes the necessary inputs of KIN more exactly.

We agreed that this is a valid concern, and clarified this section as follows (lines 405-6):

Instead, our method detects long ROH based on just the expected heterozygosity p_0, which can

be estimated from a small number of unrelated individuals..

2. Figure 1 is large and unclear. The colors of the top panel are not labeled or described in the figure legend. The figure could be improved by adding a schematic of the parental chromosomes.

We clarified this figure by introducing the colors in an additional schematic of the parental chromosomes, and made the figure more compact. The legend now reads

IBD sharing between siblings without and with runs of homozygosity (ROH). (A) Schematic of chromosomes for two parents (Par1, Par2). (B) Schematics of recombinant chromosomes of two children (Sib1, Sib2). (C) Expected differences between Sib1 and Sib2 (p) along the chromosome. In both cases, p can take values of p_0, p_1 or p_2, which are the expected proportion of differences in IBD states 0,1 and 2, respectively. (D-F): Same as A-C, except parent 1 is assumed to be homozygous.

3. In general, the paper reads as though it were born of a technical note describing the method and has not evolved far from that. This style may suit many readers. But I found it to be overly reliant on mathematical descriptions. Many readers may appreciate more verbiage describing the rationale for the equations. A well-crafted descriptive sentence or two introducing an equation is an invitation to understand it. A lone equation, with only a character list of variables is a warning to stay away.

We agree that more verbal descriptions are often easier to follow, but chose this technical writing style because we felt it was necessary to correctly describe our model. We apologize that this makes following the manuscript more difficult.

4. In section 2.2, Dw is variously described as "the expected number of pariwise differences", "the number of sites that are different" (i.e., observed differences), and a number that "needs to be estimated". From later context, it's clear that Dw is a number that is observed from analysis of the data. Please don't make the reader work so hard to understand! Clean up this section.

We agree that this section was poorly worded. We now clarified this in line 114:

For high-coverage data, D_w can be directly obtained by comparing genotypes, but for low-coverage samples, D_w needs to be estimated from the sequencing data. The simplest approach is to randomly-sample a read from each position ..

5. Concerning the equation around line 164, introducing p(Zw,Hw), should there not be a term for p4, i.e., the case where the two samples are from the same individual and that individual happens to be in a ROH?

In the case described, all haplotypes will be identical, which was our case p3. We agree that it is more natural to think of the subscript as the number of comparisons between identical chromosomes, and relabeled p3 to p4. We also expanded the section leading to eq 6 (line 183 and following) accordingly.

6. Setting p3 to p2/2 (line 167) is noted with unapologetic arbitrariness. Is it that p3 is expected to be half of p2 and p2 is expected to be estimated more accurately than p3 can be? If so - or something like that - please encourage and assist the interested reader.

As detailed on lines 148-9, p3 is expected to be zero, and we believe this confusion was caused by the misunderstanding we addressed in point 5 above. We clarified our explanation on line 152-5: As explained above, we would expect p_4 to be zero. However, as we do all our calculations in large windows, the start/end positions of windows may not coincide with that of ROH tracts, and we found that we obtain better results by setting p_4 = p_2 / 2, to take into account that many windows will only partially have four comparisons between identical chromosomes.

7. The beta binomial model appears to be appropriate and useful. The motivation for it is that "genetic linkage" increases correlation of site behavior in linked windows. I have my doubts about this. It does not really matter. Whatever the cause of the increased variance, it is handled. However, the authors could devise an experiment (same data, randomized site location, e.g.) to determine if the increased variance is attributable to linkage.

We have removed the line mentioning that the increased variance is due to correlation between sites that is attributable to genetic linkage, since this is not important for this paper.

8. Section 2.6, "Because the models we test are not nested..." - it's not clear what "nested" means in this context. Does "nested" mean "independent" or "non-overlapping" or something like that. Perhaps nested models is a common term describing model relationships. I, however, am not familiar with it and would benefit from a less jargony description.

The reviewer is correct in inferring that comparisons between nested models are well-studied, and it is known, for example, that the likelihood ratio of nested models is chi-squared distributed. However, since our models are not nested, we cannot apply this theory. On line 199-201 we expand with a brief summary of what nested means: Since we compare models where the parameters are not subset of each other, standard likelihood-ratio theory for nested models cannot be used to obtain confidence intervals.

9. Figure 2: I fail to understand why the third panel, "Identical", does not show increased likelihood in sections where the sibs co-inherit the same chromosome from both parents. Similarly, in the parent-child model, there should be a likelihood bump when the siblings have one chromosome identical by descent.

As mentioned in the legend of figure 2, the y-axis denotes the IBD state, and the number on top-left denotes the log likelihood of the model. The third panel “identical” always predicts IBD state of 2 in all windows because the transition matrix for identical relatedness predicts that all genomic windows should be in IBD state 2. However, this is not a good fit for siblings, and hence the log likelihood value for this model is lower than the siblings model. Similarly, parent-child model predicts IBD state 1 in all windows, and has a lower log likelihood compared to siblings model.

We expanded the discussion of this figure in the main text (lines 219-223):

The models for identical, parent-child or unrelated relationships allow for just one IBD state, resulting in a flat line and low likelihood for this data. The other three cases all allow for different IBD-states, but the siblings-model predictions match true IBD states the most, as reflected by the highest log-likelihood and the close correspondence of the inferred and true IBD states.

0. Figure 2: This figure needs a title. That title should have the phrase "simulated siblings" in it. As it is, the reader needs to get to the third line of the caption to learn what the figure is about.

We have now added a title for figure 2: KIN-HMMs applied to simulated siblings.

1. ROH detection section - the accuracy of the ROH HMM is reported in "bias" and "variance". These metrics are non-intuitive. Why not report the accuracy in terms of sensitivity & specificity at some reasonable cutoff?

We agree that sensitivity and specificity is a more natural analysis in this case, and have redone this analysis. We present the results in table 2, and explain this analysis in lines 287-95: A systematic evaluation of the performance of the ROH-HMM is given in table 2. For the purpose of this analysis, we classified all windows that were at least 20% homozygous as ROH, and the remainder as non-ROH. Likewise, we classified all windows with a posterior probability of ROH of at least 20% as ROH. We used these cases to compute sensitivity (Se) and specificity (Sp). In the control case (simulations with no ascertainment, contamination, or ROH) we see that the specificity remains above 0.99 for all coverages. In the cases where we simulate ROH, we find that sensitivity decreases from 97% at 4x coverage to less than 65% at 0.05x coverage, but specificity remains high at above 0.96 for all cases, suggesting that the number of erroneously called ROH segments is low.

2. Line 327, as written, it's not clear if the "(<3%)" describes the level of contamination or the true positive rate.

We have reworded this sentence in lines 322-3: To investigate the impact of contamination, we performed simulations where we added up to 3% contamination to some individuals.

3. The authors chose to report their relatedness result as the LLR between the two best models. As they point out, there are discrete models of relatedness. For example, one can be 50% or 100% IBD, but not 75%. As relatedness becomes more distant, the distance between them decreases. Thus, under this framework, it is necessarily more difficult to distinguish between competing models of distant relatedness than it is between competing models of more close relatives. That is, even with perfect data, the LLR comparing 4th versus 5th degree relatives will be lower than the LLR between 1st and 2nd degree relatives. For this reason, it may be possible to derive a maximum likelihood or other classifier that takes the likelihoods of all models.

We agree that it would be nice to have a proper ML classifier. However, as laid out in our response to point 8, we don’t think ML-theory applies to this case which is why we chose to use this approximate scheme that we show, using simulations, works reasonably well to classify up to 3-rd degree relationships.

**Second round of review**

**Reviewer 1**

The updated manuscript is much improved and the authors have addressed most of my previous points. I only have minor lurking comments, which the authors can ignore if they disagree.

Thank you for the clarification regarding the variability in IBD states in Figure 4. These sentiments would be nice to include in the paper. It may also be worth emphasizing that this is not the overall classification accuracy of KIN, but rather its per-window performance in classifying IBD states.

The caption of Figure S13 says “For this analysis we varied the contamination level in one individual from 0.5% to 5.5% while keeping contamination estimates for everyone else constant.” Should this say “we varied the _estimated_ contamination level”? Since the previous sentence says that the true contamination level is 2.5%.

**Reviewer 2**

The authors have adequately addressed all issues I had with the manuscript.
